# Psychometric properties of the Persian version of the self-regulated learning scale in clinical nursing practice for nursing students: A methodological study

**DOI:** 10.1371/journal.pone.0358790

**Published:** 2026-09-25

**Authors:** Ali Bazzi, Zahra Dizaji, Zahra Mozaffari, Reza Nemati-Vakilabad

**Affiliations:** 1 Phd in nursing, Clinical Research Development Unit (CRDU), Baqiyatallah al-Azam Hospital, Golestan University of Medical Sciences, Aliabad-e katul, Iran; 2 Department of Nursing, Gonbad Kavous School of Nursing, Golestan University of Medical Sciences, Gonbad Kavous, Iran; 3 Students Research Committee, School of Nursing and Midwifery, Ardabil University of Medical Sciences, Ardabil, Iran; 4 Department of Emergency Nursing, School of Nursing and Midwifery, Ardabil University of Medical Sciences, Ardabil, Iran; 5 Department of Medical-Surgical Nursing, School of Nursing and Midwifery, Guilan University of Medical Sciences, Rasht, Iran; Dayeh University, TAIWAN

## Abstract

**Background:**

Self-regulated learning is essential for nursing students to navigate complex clinical environments and achieve academic and professional success. This study aimed to validate the Persian version of the Self-Regulated Learning Scale for Clinical Nursing Practice (SRLS-CNP-P) among Iranian nursing students.

**Methods:**

A methodological study was conducted using stratified random sampling, involving 360 undergraduate nursing students from Guilan University of Medical Sciences in Iran, following COSMIN guidelines. The scale underwent forward-backward translation, and its psychometric properties were evaluated through several methods: face and content validity, construct validity, and convergent and discriminant validity. Additionally, the reliability of the scale was assessed through measures of internal consistency and test-retest reliability.

**Results:**

The SRLS-CNP-P demonstrated strong face and content validity (I-CVI = 0.8–1.0; $K^{*}$ = 0.79–1.0). EFA revealed a five-factor structure explaining 81.49% of the total variance, which CFA confirmed with excellent fit indices. Convergent validity was supported (CR = 0.828–0.913; AVE = 0.548–0.839), and discriminant validity was established (HTMT < 0.85). Reliability was robust, with Cronbach’s α = 0.723–0.911, McDonald’s $\omega_{\text{t}}$ = 0.731–0.920, and ICC = 0.783–0.872 for test-retest reliability.

**Conclusion:**

The SRLS-CNP-P demonstrated strong psychometric properties among undergraduate nursing students at the participating institution, providing preliminary evidence for its use in this population. Future research is needed to confirm its applicability across other Persian-speaking nursing student populations. It may help educators evaluate and enhance students’ clinical learning strategies, supporting academic and professional development.

## Introduction

Nursing students are expected to graduate as lifelong learners who continuously integrate evolving knowledge into their practice, remain open to professional development, and deliver high-quality patient care [1]. Clinical learning environments are inherently complex and often expose students to significant stress as they engage with real-world patient care scenarios [2,3]. Consequently, students must consistently evaluate their performance, regulate their learning processes, and adapt to dynamic clinical settings [4,5]. This continuous cycle of self-assessment, learning management, and environmental adaptation is essential for navigating the challenges of clinical education [2,6].

Self-regulation is a fundamental competency for nursing students, encompassing the ability to control thoughts, emotions, and behaviors to achieve academic and professional success. This skill is particularly critical within the rigorous demands of nursing education, where students must balance intensive coursework with clinical responsibilities. Effective self-regulation not only enhances academic achievement but also improves patient care and personal well-being, underscoring its significance in nursing education frameworks [7,8].

The theoretical foundations of self-regulation in nursing education are rooted in various learning theories. Zimmerman’s self-regulated learning (SRL) theory highlights metacognition, motivation, and strategic action as essential components, conceptualizing SRL as a cyclical process involving forethought (planning and goal-setting), performance (monitoring and strategy implementation), and self-reflection (evaluation and adaptation) [9]. Pintrich’s model similarly emphasizes four phases of SRL: planning and activating, monitoring, control, and reflection, across cognitive, motivational/affective, behavioral, and contextual domains [10]. Panadero’s comprehensive review of SRL models further underscores SRL’s multidimensional nature, encompassing cognitive, metacognitive, motivational, behavioral, and increasingly social processes [11]. Additional frameworks, such as cognitive and behavioral learning theories, further elucidate how self-regulation can be cultivated through active engagement and deliberate skill development [12].

The Self-Regulated Learning Scale for Clinical Nursing Practice (SRLS-CNP) was specifically designed to capture selected motivational and strategic aspects of learning that are particularly relevant to clinical nursing education. The five factors of the scale map onto key components of SRL theory as follows: Intrinsic and Achievement Motivation align with the motivational phase of Pintrich’s model, reflecting the driving force for engagement in clinical learning; Synthesized Knowledge and Nursing Skills corresponds to cognitive strategy use; Multidimensional Thinking reflects metacognitive processes including critical reflection and perspective-taking; and Effort Control represents behavioral persistence in the face of challenges [3]. Importantly, the scale does not claim to capture the full breadth of SRL as defined in comprehensive theoretical models—such as the full cyclical processes of planning, monitoring, self-evaluation, and regulation based on feedback—but rather focuses on specific motivational and strategic dimensions that are particularly salient in the clinical learning context. This targeted approach is consistent with the scale’s original development by Iyama and Maeda (2018), who prioritized practical clinical relevance over comprehensive theoretical coverage [3].

The Self-Regulated Learning Scale for Clinical Nursing Practice (SRLS-CNP) has been developed and validated as a reliable instrument for assessing nursing students’ self-regulated learning in clinical settings. This scale incorporates subscales measuring motivation and learning strategies, providing a comprehensive evaluation of self-regulation in clinical education [13]. With strong reliability and validity, the SRLS-CNP facilitates the assessment of self-regulated learning across different academic years, offering valuable insights into students’ developmental progress throughout nursing education [3].

Nursing education has undergone significant transformations since 2019, driven by factors including the COVID-19 pandemic [14]. This crisis prompted a shift to online teaching methods and emphasized critical care [15], alongside advancements in technology [16] and an increased reliance on simulation and AI tools [17]. Furthermore, changes in students’ social routines and a move towards self-regulated learning have led to more flexible, interactive teaching approaches, making nursing education more adaptable to the evolving demands of healthcare [18].

In this context, nurse educators play a pivotal role in promoting SRL, grounded in self-regulation theory, by modeling and teaching strategic learning practices [19]. These methods empower students to take ownership of their education, enhancing skills such as time management, goal setting, and progress monitoring [20]. However, despite growing recognition of self-regulation’s importance in nursing education, a significant research gap remains regarding nursing students’ self-regulatory behaviors [21].

Understanding SRL is particularly important for nursing students, as it directly affects their academic performance, clinical competence, and professional growth [21,22]. A thorough literature review indicates that effective self-regulatory practices are linked to improved learning outcomes and enhanced adaptation to the challenges of nursing education [23,24]. Nevertheless, in the Iranian context, the lack of culturally relevant assessment tools hinders our understanding of students’ self-regulatory behaviors.

Existing scales for assessing self-regulated learning may fall short in addressing the unique cultural and educational challenges faced by Iranian nursing students. Adapting the SRLS-CNP scale, originally developed by Iyama and Maeda (2018) in Japan [3], can fill this critical gap. This 16-item scale comprises two subscales: Motivation (including intrinsic motivation and achievement motivation) and Learning Strategies (including synthesized knowledge and nursing skill, multidimensional thinking, and effort control).

By adapting the SRLS-CNP, we aim to create a reliable and culturally relevant tool that fits the specific context of Iranian nursing students. This adaptation is essential for developing targeted educational interventions and promoting a culture of self-regulation tailored to the unique challenges these students face. A rigorous psychometric evaluation of this adapted scale will determine its reliability and validity, enabling precise assessments and effective support to enhance self-regulatory skills in nursing education. Hence, this study aims to advance educational strategies in Iranian nursing by providing insights into self-regulated learning and its assessment, ultimately improving student outcomes and professional development.

## Materials and methods

### Study design

This methodological study evaluated the psychometric properties of SRLS-CNP-P among nursing students at Guilan University of Medical Sciences from June to December 2024. The study followed COSMIN guidelines for content validity, structural validity, internal consistency, and reliability [25]. However, not all COSMIN measurement properties were assessed; measurement error, hypothesis testing against related constructs, responsiveness, and criterion validity were beyond the scope of this validation study.

### Setting and sample

The study was conducted on nursing students at the Shahid Beheshti School of Nursing and Midwifery in Rasht, Iran. The sampling frame consisted of 520 eligible undergraduate nursing students across three academic years (second year: *n* = 175; third year: *n* = 168; fourth year: *n* = 177).

We recruited a total sample of 360 undergraduate nursing students through stratified random sampling by academic year. Within each stratum, we selected participants using computer-generated random numbers (SPSS version 24; RV.UNIFORM). We determined the sample size using a two-tiered approach to meet the distinct requirements of EFA and CFA. For Exploratory Factor Analysis (EFA), we followed the conventional subject-to-item ratio of 10:1 (16 items × 10 = 160 participants) to ensure adequate power for initial factor extraction [26]. For Confirmatory Factor Analysis (CFA), we intentionally recruited an additional 200 participants (total N = 360) to exceed the minimum threshold of 200 recommended for CFA [27], as larger samples enhance model stability, mitigate convergence issues, and improve the reliability of fit indices [28,29]. To account for potential attrition, we initially approached 400 eligible participants (stratified evenly across academic years) and achieved a 90% response rate (N = 360). The number approached and responding per stratum was: second year: approached 140, responded 120 (85.7%); third year: approached 130, responded 110 (84.6%); fourth year: approached 130, responded 130 (100%). Reasons for non-participation among the 40 students included: lack of interest/time (*n* = 22), scheduling conflicts (*n* = 10), personal reasons (*n* = 5), and incomplete questionnaires (*n* = 3) [30].

The study sample comprised undergraduate nursing students actively enrolled in clinical rotations at Shahid Beheshti School of Nursing and Midwifery in Rasht who had completed at least two semesters of clinical training, ensuring adequate exposure to clinical learning environments. Participants needed to demonstrate willingness to complete both initial and follow-up assessments for reliability testing. Exclusion criteria systematically removed potential sources of bias by excluding students with interrupted clinical training due to medical leave or course withdrawals, those who had not completed fundamental clinical courses, individuals unavailable for the required retesting interval, and cases with substantial missing data (>10% incomplete responses). No participants were excluded due to missing data, as all 360 collected questionnaires were fully completed. The 3 incomplete questionnaires identified among the 40 non-participants were not included in the final sample, as they were part of the initial approach but did not meet the inclusion criteria. Since all 360 retained participants had complete data, no imputation methods (e.g., mean substitution, multiple imputation) were required.

### Self-Regulated Learning Scale in Clinical Nursing Practice (SRLS-CNP)

The Self-Regulated Learning Scale in Clinical Nursing Practice (SRLS-CNP) was developed by Iyama and Maeda (2018) in Japan as a multidimensional assessment tool specifically designed to measure self-regulated learning behaviors in clinical nursing education [3]. Before initiating this study, we obtained formal written permission from the original developers (Iyama & Maeda) to use, adapt, and validate the instrument in our cultural and educational context. This permission included explicit consent for any necessary linguistic or contextual modifications while retaining the scale’s core psychometric properties.

This 16-item instrument employs a 5-point Likert-type response format ranging from “strongly disagree” [1] to “strongly agree” [5], with total scores potentially ranging from 16 to 80, where higher scores indicate greater utilization of self-regulated learning strategies (S1 File). The scale comprises two distinct but interrelated subscales: The Motivation subscale and the Learning Strategies subscale. The Motivation subscale consists of two factors: Intrinsic Motivation (4 items), which assesses the inherent interest in clinical learning, and Achievement Motivation (3 items), which measures performance-oriented goals. The Learning Strategies subscale includes three factors: Synthesized Knowledge and Nursing Skills (5 items), which evaluates the integration of theoretical and practical knowledge; Multidimensional Thinking (2 items), which assesses critical reflection; and Effort Control (2 items), which measures persistence in challenging clinical situations. Iyama and Maeda’s scale development demonstrated strong psychometric properties in the Japanese context, with reported Cronbach’s α coefficients ranging from 0.711 to 0.838 for subscales, establishing it as a theoretically grounded tool for assessing clinically relevant self-regulated learning processes.

### Translation procedure

The scale was initially developed in Japan [3] but was translated into English by the developers for international use. Our adaptation process began with this authorized English version to maintain alignment with existing validations. The SRLS-CNP underwent cross-cultural adaptation from English to Persian. Two bilingual nursing education experts independently forward-translated the original English version into Persian. A reconciliation panel of three additional experts resolved translation discrepancies through structured discussion. The harmonized Persian version was then back-translated to English by two independent translators unfamiliar with the original scale (S2 File). An expert committee, including two nursing educators and one linguist, systematically evaluated all versions to ensure semantic, idiomatic, experiential, and conceptual equivalence.

### Psychometric testing

#### Face validity.

The face validity of the SRLS-CNP-P was assessed using a two-stage qualitative and quantitative approach. In the qualitative phase, 20 nursing students were purposively selected to evaluate each item’s clarity, relevance, and appropriateness to the target construct. Participants provided feedback on wording, comprehensibility, and structural coherence, leading to minor modifications to enhance precision and cultural adaptation. Qualitative feedback was systematically recorded and reviewed; all items were judged relevant and appropriate, with minor phrasing adjustments to improve naturalness in Persian (e.g., simplifying complex sentences, using more familiar clinical terminology).

For the quantitative assessment, the Item Impact Score (IIS) was calculated to determine the perceived significance of each item. The nursing students rated the Importance of each statement on a 5-point Likert scale (1 = not important to 5 = extremely important). The IIS was derived using the formula:


$$\begin{array}{c} \text{IIS}=\text{Frequency }(\%)\times \text{Importance} \end{array}$$

Frequency (%) reflected the proportion of respondents assigning a rating of 4 or 5, and *Importance* denoted the mean score of each item. All items with an IIS ≥ 1.5 were retained, confirming their suitability for inclusion in the final scale [31].

#### Content validity.

To ensure the relevance, clarity, and theoretical alignment of the SRLS-CNP-P, content validity was evaluated through qualitative expert review and quantitative indices. A panel of 10 experts was purposively selected based on the following criteria: holding a doctoral degree in nursing education, clinical nursing, or psychometrics; having at least 5 years of experience in nursing education or clinical practice; and expertise in instrument development or psychometric evaluation. The panel consisted of 5 nursing educators (with PhDs in Nursing Education), 3 clinical nursing specialists (with MSc in Nursing and >10 years of clinical experience), and 2 psychometricians. Their mean years of experience were 12.4 years (SD = 4.2). The experts assessed each item’s representativeness, clarity, and fit with self-regulated learning (SRL) in clinical nursing education. Their qualitative feedback guided refinements to wording and conceptual precision to better capture the intended construct [32]. Content comprehensiveness was assessed by asking experts whether the scale’s items adequately covered all important aspects of self-regulated learning in clinical nursing practice as defined by the theoretical framework [25]. Experts confirmed that the items comprehensively addressed the key domains (motivation and learning strategies), with no major content gaps identified. Minor concerns regarding item wording were resolved through iterative revisions.

For quantitative validation, two key indices were computed. The Item Content Validity Index (I-CVI) was determined by having experts rate each item’s relevance on a 4-point scale, ranging from 1 (not relevant) to 4 (highly relevant). The I-CVI score for each item was calculated as the proportion of experts assigning ratings of 3 or 4, and items scoring ≥ 0.78 were retained due to their strong relevance. Modified Kappa ($K^{*}$) statistics were also calculated for each item to adjust for potential chance agreement among raters. $K^{*}$ values exceeding 0.74 indicated excellent inter-expert consensus, further supporting the scale’s content validity [33]. Scale-level content validity was assessed using two indices: the average scale-level content validity index (S-CVI/Ave), calculated as the mean I-CVI across all items, with a recommended threshold of ≥ 0.90; and the universal agreement scale-level content validity index (S-CVI/UA), defined as the proportion of items rated as relevant by all experts, with an acceptable threshold of ≥ 0.80 [33].

#### Construct validity.

The assessment of construct validity began with an Exploratory Factor Analysis (EFA) using Maximum Likelihood Estimation (MLE) with Promax rotation, chosen to accommodate anticipated correlations among factors. The analysis applied multiple criteria for factor extraction, including eigenvalues greater than 1.0, a review of scree plot inflection points, and parallel analysis using 1000 random datasets generated with the same number of variables and sample size as the original data (*n* = 160, 16 items). In parallel analysis, factors are retained when eigenvalues from the actual data exceed the corresponding 95th percentile eigenvalues from randomly generated data, providing a more accurate and conservative approach to factor retention than the eigenvalue > 1.0 rule alone. The adequacy of the sample was confirmed through the Kaiser-Meyer-Olkin (KMO) measure and Bartlett’s test of sphericity. Items were retained when they demonstrated a primary factor loading of ≥ 0.50 and all secondary (cross) loadings were < 0.40. The factor correlation matrix resulting from the Promax rotation was also examined to assess the relationships among the extracted factors. Extraction converged within a maximum of 25 iterations, and the number of iterations required for convergence was recorded [34].

Given the ordinal nature of the 5-point Likert-scale items, we first evaluated the data’s distributional properties. We examined item-level descriptive statistics, including means, standard deviations, skewness, and kurtosis, to assess univariate distributions. All items demonstrated acceptable skewness (ranging from −0.58 to 0.33) and kurtosis (ranging from −0.52 to 0.42), well within the acceptable range of ±2 [35], supporting univariate normality. However, to account for the ordinal nature of the data and to ensure robust parameter estimation, we employed two complementary approaches for the CFA. First, we used Maximum Likelihood (ML) estimation with bootstrapping (1000 bootstrap samples) to obtain robust standard errors and confidence intervals, as ML with bootstrapping is relatively robust to mild violations of normality when sample sizes are adequate (*n* = 200) [36]. Second, we conducted a sensitivity analysis using the Weighted Least Squares Mean and Variance Adjusted (WLSMV) estimator, which is specifically designed for ordinal data and utilizes polychoric correlations [37,38]. In Confirmatory Factor Analysis (CFA), the hypothesized five-factor structure was tested using both ML estimation with 200 independent participants and WLSMV estimation as a sensitivity analysis, applying established thresholds for model evaluation: acceptable factor loadings ≥ 0.50, critical ratios (C.R.) > 1.96 (*p* < 0.05), and goodness-of-fit indices including absolute fit (χ²/df < 3 acceptable, < 2 excellent; RMSEA < 0.08 acceptable, < 0.05 excellent with 90% CI upper limit < 0.10), incremental fit (CFI > 0.90 acceptable, > 0.95 excellent; IFI > 0.90; TLI > 0.90), and parsimony indices (PGFI > 0.50; PCFI > 0.50) [39] for the ML estimation. For the WLSMV estimation, we evaluated model fit using the robust chi-square (χ²_robust), RMSEA, CFI, and TLI, with the same threshold criteria [38].

We defined the five-factor model according to its original structure [3], allowing for correlations among all factors. We did not include correlated residuals or make any post-hoc modifications because the initial model already showed an excellent fit. To assess the hierarchical structure, we tested four competing models: (1) a one-factor model; (2) a two-factor model (Motivation and Learning Strategies); (3) a first-order five-factor model (as hypothesized); and (4) a higher-order model with two second-order factors. We compared the models using chi-square difference tests and information criteria, including AIC and BIC [27].

#### Convergent and discriminant validity.

Convergent validity was assessed through two primary criteria: composite reliability (CR) values were calculated with acceptable thresholds set at > 0.70 for adequate reliability, while average variance extracted (AVE) values were evaluated against the > 0.50 benchmark, indicating sufficient item convergence on each latent construct [40]. For discriminant validity, three complementary approaches were employed: The Fornell-Larcker criterion requiring each construct’s AVE to exceed its squared correlations with other constructs, comparison of Average Shared Variance (ASV) and Maximum Shared Variance (MSV) against AVE values, and Heterotrait-Monotrait Ratio (HTMT) analysis with a conservative threshold of < 0.85 and bootstrapped 95% confidence intervals (5000 bootstrap samples) [41].

#### Reliability.

The internal consistency of the SRLS-CNP-P was assessed using multiple indices: Cronbach’s alpha (α), a classic measure of item interrelatedness; McDonald’s omega ($\omega_{\text{t}}$), which accounts for factor structure and is less biased with non-tau-equivalent items; Coefficient H, reflecting the construct’s maximal reliability in a multidimensional scale; and the Greatest Lower Bound (GLB), providing the strictest estimate of reliability by considering shared variance. All values exceeded 0.70, confirming strong internal consistency [42–44]. For the two-item factors (Multidimensional Thinking and Effort Control), we additionally report inter-item correlations and Spearman-Brown coefficients [45,46]. Original developers [3] conceptualized the scale as comprising two broader subscales with five first-order factors and did not explicitly validate a single total score. Accordingly, we focus our interpretation on factor and subscale scores. Total-scale coefficients are provided for descriptive purposes only.

To assess the temporal stability of the SRLS-CNP-P, we calculated the Intraclass Correlation Coefficient (ICC) with a 95% confidence interval (CI) following a 2-week retest administered to 30 nursing students. For the test-retest reliability analysis, we selected a subsample of 30 participants using simple random sampling from the original 360 participants who completed the initial assessment. No significant differences were found between the retest subsample and the remaining participants in terms of age (t = 0.42, *p* = 0.675), gender (χ² = 0.18, *p* = 0.671), or academic year (χ² = 0.94, *p* = 0.625), indicating that the retest sample was representative of the overall study population. We calculated the ICC using a two-way mixed-effects model to assess absolute agreement, and reported single-measure ICC values. The ICC surpassed the recommended threshold of 0.75, indicating good to excellent test-retest reliability [47].

### Statistical analysis

We performed all statistical analyses using two specialized software packages (S3 File). For descriptive statistics (means, standard deviations, and distribution properties (skewness and kurtosis)) and preliminary reliability analysis, we employed IBM SPSS Statistics for Windows, version 24 (IBM Corp., Armonk, NY, USA). For psychometric testing, including confirmatory factor analysis (CFA) and structural equation modeling (SEM), we used IBM SPSS AMOS Graphics (Version 24; IBM Corp.). McDonald’s omega ($\omega_{\text{t}}$), Coefficient H, and the Greatest Lower Bound (GLB) were calculated using the R package “psych” (version 2.3.6). At the same time, HTMT values were computed using the “semTools” package in R. We adopted a conventional two-tailed significance level (*p* < 0.05) for hypothesis testing throughout all analyses.

### Ethical considerations

The research was conducted in accordance with the principles outlined in the Declaration of Helsinki and adhered to national guidelines for biomedical research. Ethical approval (IR.GUMS.REC.1403.040) was obtained from the Research Ethics Committee of Guilan University of Medical Sciences. Before starting the study, all participating nursing students were thoroughly informed about the research objectives, procedures, and their rights, including voluntary participation, the freedom to withdraw at any time, and assurance of complete confidentiality regarding their information. We obtained written informed consent from all participants.

## Results

### Characteristics of the participants

The study included 360 participants, divided into samples for exploratory factor analysis (EFA, *n* = 160) and confirmatory factor analysis (CFA, *n* = 200). The overall sample had a mean age of 21.7 years (SD = 2.01) and an average GPA of 16.52/20 (SD = 3.35). The majority of participants were female (66.7%, *n* = 240), single (86.1%, *n* = 310), and distributed across academic years as follows: second year (33.3%, *n* = 120), third year (30.6%, *n* = 110), and fourth year (36.1%, *n* = 130) (Table 1).

**Table 1:** Demographic characteristics of the participants (*n* = 360).

Variable	Category	EFA (*n* = 160)	CFA (*n* = 200)	Total (*n* = 360)
Age (years), Mean (SD)		21.3 ± 1.75	22.1 ± 2.04	21.7 ± 2.01
GPA (out of 20), Mean (SD)		16.3 ± 3.58	16.64 ± 3.16	16.52 ± 3.35
Gender, *n* (%)	Male	51 (31.9%)	69 (34.5%)	120 (33.3%)
	Female	109 (68.1%)	131 (65.5%)	240 (66.7%)
Marital status, *n* (%)	Single	131 (81.9%)	179 (89.5%)	310 (86.1%)
	Married	29 (18.1%)	21 (10.5%)	50 (13.9%)
Year of study, *n* (%)	Second	62 (38.8%)	58 (29.0%)	120 (33.3%)
	Third	49 (30.6%)	61 (30.5%)	110 (30.6%)
	Fourth	49 (30.6%)	81 (40.5%)	130 (36.1%)

Item-level descriptive statistics, including means, standard deviations, skewness, kurtosis, and floor/ceiling effects, are presented in(S4 File). All items demonstrated acceptable normality with skewness values ranging from −0.58 to 0.33 and kurtosis values from −0.52 to 0.42, well within the acceptable range of ±2. No floor or ceiling effects were observed, as all item means fell within the mid-range of the 5-point Likert scale (2.42 to 3.81), and the percentage of responses at the extreme values (scores 1 and 5) ranged from 0.3% to 8.6% for floor and 1.9% to 11.4% for ceiling, all well below the 15% threshold.

### Face validity

Participants evaluated the translated items’ clarity, relevance, and cultural appropriateness in the qualitative phase. Their feedback indicated that the items were clear and relevant to self-regulated learning in clinical contexts. However, they suggested minor linguistic adjustments to make the phrasing more natural in Persian. Examples of modifications included: changing “I confirm clinical nursing skills” to “I review and verify clinical nursing skills” for greater clarity; revising “I would like to develop my way of thinking” to “I seek to expand my thinking approach” to better reflect the reflective intent; and simplifying “I try to associate what I have experienced through clinical nursing practice with the knowledge I previously acquired” to “I connect my clinical experiences with my prior knowledge” for improved readability. These changes were made without altering the original meaning of the items.

The Item Impact Score was calculated for the quantitative assessment, with scores ranging from 2.5 to 3.2 (≥ 1.5). All items exceeded the threshold, confirming their suitability for the intended construct. No items were eliminated at this stage (Table 2).

**Table 2:** Results of the face and content validity of the SRLS-CNP-P.

Items	IIS	A	I-CVI	$P_{C}$	$K^{*}$	Interpretation
1	2.5	8	0.8	0.044	0.79	Excellent
2	3.2	9	0.9	0.01	0.89	Excellent
3	2.7	9	0.9	0.01	0.89	Excellent
4	2.5	8	0.8	0.044	0.79	Excellent
5	2.7	8	0.8	0.044	0.79	Excellent
6	2.7	10	1	0.001	1	Excellent
7	2.7	9	0.9	0.01	0.89	Excellent
8	2.5	8	0.8	0.044	0.79	Excellent
9	2.5	8	0.8	0.044	0.79	Excellent
10	3.2	9	0.9	0.01	0.89	Excellent
11	2.7	9	0.9	0.01	0.89	Excellent
12	3.2	10	1	0.001	1	Excellent
13	2.5	8	0.8	0.044	0.79	Excellent
14	2.5	10	1	0.001	1	Excellent
15	2.5	8	0.8	0.044	0.79	Excellent
16	3.2	9	0.9	0.01	0.89	Excellent

**Abbreviations:** IIS, Item Impact score; A, Number agreeing on good relevance; I-CVI, Item content validity index; $P_{C}$, Probability of a chance occurrence; $K^{*}$, Modified Kappa designating agreement on relevance.

### Content validity

In the qualitative evaluation, experts examined each item for appropriateness, clarity, and relevance to self-regulated learning in clinical nursing education. Their feedback led to minor wording changes to improve item clarity while preserving the original construct meanings. Examples of modifications included: changing “I confirm clinical nursing skills before providing care” to “I verify clinical nursing skills before providing patient care”; rewording “I learn patient materials” to “I study patient-related materials”; and refining “I want to explain important points” to “I aim to explain key learning points.” All items were judged to be conceptually relevant to the target domain.

The I-CVI was calculated for the quantitative assessment. As shown in Table 2, all items demonstrated excellent content validity, with I-CVI values ranging from 0.8 to 1.0. The modified Kappa statistic ($K^{*}$) values adjusted for chance agreement ranged from 0.79 to 1.0, confirming excellent content validity for all scale items. The S-CVI/Ave was 0.91, and S-CVI/UA was 0.75.

### Construct validity

The results of EFA showed excellent factorability (KMO = 0.87, Bartlett’s test of sphericity (χ² = 1284.32, *p* < 0.001)), revealing five clear factors explaining 81.49% of the variance. All three retention methods supported a five-factor solution. The eigenvalue > 1.0 criterion identified five factors (eigenvalues: 3.117, 2.871, 2.512, 2.341, and 2.198; the sixth eigenvalue was 0.987). Scree plot inspection revealed a clear inflection point after the fifth factor, with the curve leveling off thereafter, consistent with a five-factor structure. Parallel analysis confirmed the retention of five factors, as the eigenvalues obtained from the actual data exceeded the 95th percentile random eigenvalues for the first five factors (actual eigenvalues: 3.117, 2.871, 2.512, 2.341, 2.198 vs. random 95th percentile eigenvalues: 1.562, 1.488, 1.421, 1.365, 1.298). For the sixth factor, the actual eigenvalue (0.987) fell below the random 95th percentile eigenvalue (1.245), supporting the five-factor solution. This convergence between all three retention methods strengthens confidence in the factor retention decision.

The pattern matrix from the Promax rotation demonstrated that all items loaded strongly on their respective factors, with primary loadings ranging from 0.75 to 0.85. All secondary loadings (cross-loadings) were below 0.40, confirming the clarity of the factor structure. Table 3 presents the complete pattern matrix, including primary loadings. The factor correlation matrix from the Promax rotation (Table 6) revealed moderate correlations among factors (ranging from 0.021 to 0.414), supporting the use of Promax rotation, which allows correlated factors. These correlations were consistent with those obtained from the CFA model, further confirming the interrelated yet distinct nature of the five factors. The extraction algorithm converged successfully after 8 iterations, indicating stable factor extraction. The structure showed good communalities (0.63–0.77) and conceptually coherent patterns, supporting the scale’s validity for measuring nursing students’ self-regulated learning in clinical settings (Table 3).

**Table 3:** The results of the exploratory factor analysis of the SRLS-CNP-P (*n* = 160).

Factor	Items	Mean ± SD	Factor loading	*h* ^*2*^	λ	% of variance
Intrinsic motivation	**Motivation subscale**					
	1. I like clinical nursing practice even though it is difficult.	3.62 ± 0.71	0.812	0.723	3.117	19.48%
	2. I like clinical nursing practice even if I cannot get good grades.	3.68 ± 0.74	0.841	0.719		
	3. I like clinical nursing practice because I can learn new things.	3.81 ± 0.73	0.825	0.751		
	4. I am satisfied with myself because I am working hard during clinical nursing practice.	3.59 ± 0.72	0.793	0.672		
Achievement motivation	5. It is important for me to get good grades in clinical nursing practice.	3.72 ± 0.73	0.832	0.769	2.871	17.94%
	6. I want to get better grades than my classmates for clinical nursing practice.	3.75 ± 0.56	0.846	0.741		
	7. I would like to demonstrate my ability to others by getting good grades in clinical nursing practice.	3.08 ± 0.51	0.819	0.641		
Synthesized knowledge and nursing skills	**Learning strategies subscale**					
	8. I confirm clinical nursing skills before providing care to patients.	3.74 ± 0.48	0.839	0.628	2.512	15.70%
	9. Using my own words, I want to explain important points I have learned in my clinical nursing practice.	2.42 ± 0.75	0.752	0.659		
	10. I think about ways to implement care tailored to individual patient needs.	3.72 ± 0.43	0.818	0.702		
	11. I learn patient materials before clinical nursing practice.	3.45 ± 0.62	0.789	0.698		
	12. I practice nursing skills by myself during a clinical nursing practice period.	3.52 ± 0.58	0.814	0.721		
Multidimensional thinking	13. I would like to develop my way of thinking based on what I have learned through clinical nursing practice.	3.61 ± 0.55	0.831	0.702	2.341	14.63%
	14. I wonder if there are other ways of thinking about what I have learned through clinical nursing practice.	3.48 ± 0.59	0.819	0.689		
Effort control	15. I make an effort to understand difficult concepts I have learned from clinical mentors and instructors.	3.57 ± 0.61	0.802	0.678	2.198	13.74%
	16. I try to associate what I have experienced through clinical nursing practice with the knowledge I previously acquired.	3.62 ± 0.57	0.814	0.691		

**Abbreviations:** *h*^*2*^, Communalities; λ, Eigenvalue.

**Fig 1:**
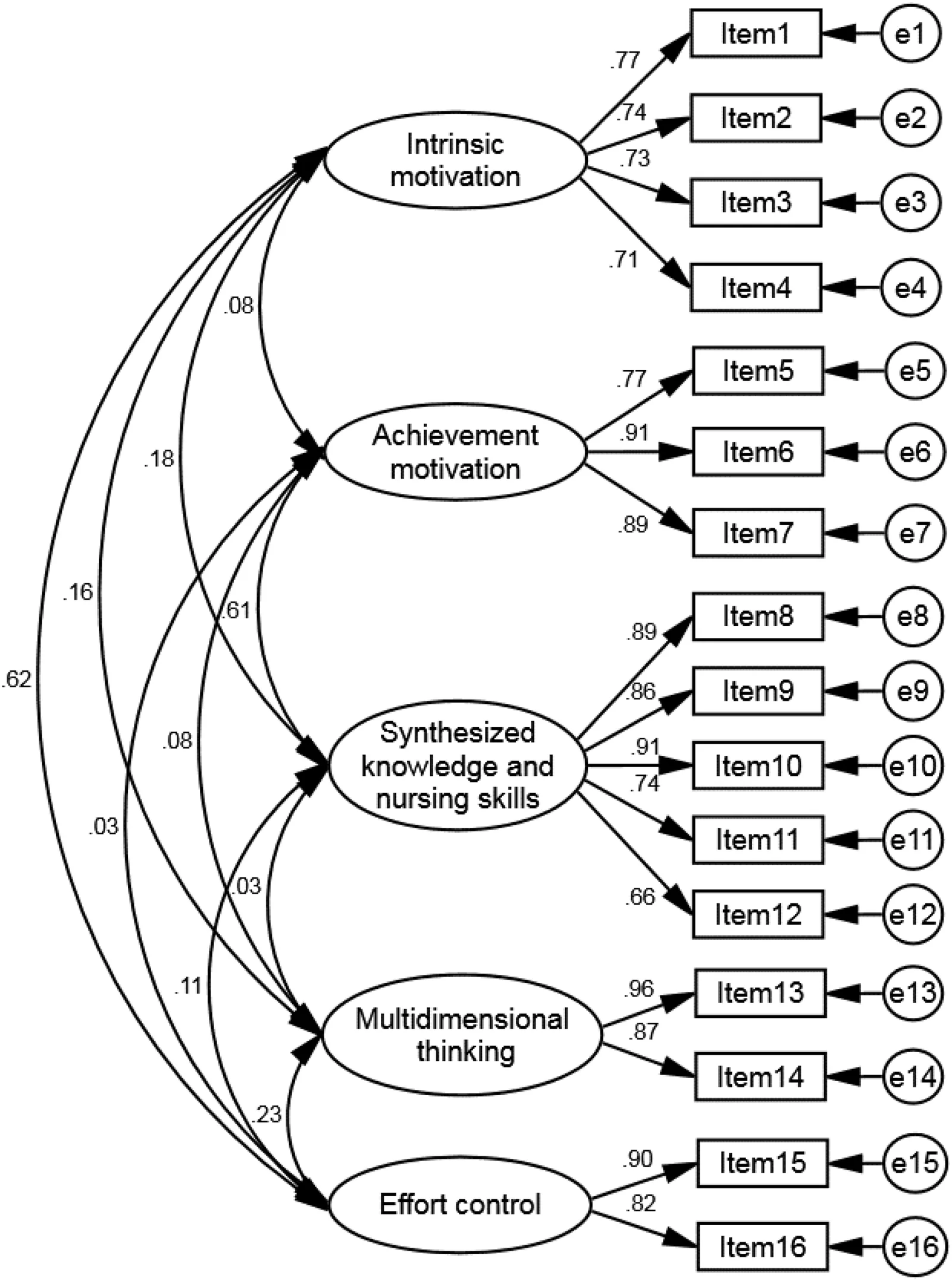
The final model of the first-order CFA of the SRLS-CNP-P (*n* = 200).

The results of CFA demonstrated excellent model fit, with all factor loadings ranging from 0.66 to 0.96, indicating strong relationships between items and their respective latent constructs (Fig 1). The critical ratio values ranged from 5.698 (S.E. = 0.164) to 21.485 (S.E. = 0.042), demonstrating statistically significant factor loadings (*p* < 0.001) (Table 4). The model fit indices revealed χ² = 139.739 with 94 degrees of freedom (χ²/df = 1.487), RMSEA = 0.044 (90% CI: 0.035–0.053), SRMR = 0.041, GFI = 0.935, CFI = 0.980, TLI = 0.975, RFI = 0.928, and IFI = 0.981, all exceeding recommended thresholds for good model fit. The parsimony indices (PGFI = 0.646, PCFI = 0.768) and close-fit test (PCLOSE = 0.725) further confirmed the model’s appropriate balance between complexity and fit.

**Table 4:** Standardized factor loadings, standard errors, and critical ratios for the CFA model of the SRLS-CNP-P (*n* = 200).

Factor	Item	Standardized Loading	S.E.	C.R.	*p*-value
Intrinsic motivation	1	0.77	0.082	9.512	< 0.001
	2	0.74	0.071	11.972	< 0.001
	3	0.73	0.075	10.933	< 0.001
	4	0.71	0.095	7.474	< 0.001
Achievement motivation	5	0.77	0.068	12.353	< 0.001
	6	0.91	0.059	14.915	< 0.001
	7	0.89	0.088	8.523	< 0.001
Synthesized knowledge and nursing skills	8	0.89	0.064	13.438	< 0.001
	9	0.86	0.105	6.286	< 0.001
	10	0.91	0.073	11.233	< 0.001
	11	0.74	0.079	10.001	< 0.001
	12	0.66	0.069	12.174	< 0.001
Multidimensional thinking	13	0.96	0.071	11.690	< 0.001
	14	0.87	0.076	10.658	< 0.001
Effort control	15	0.90	0.085	9.059	< 0.001
	16	0.82	0.078	10.256	< 0.001

**Abbreviations:** S.E., Standard Error; C.R., Critical Ratio.

To assess the robustness of our findings to the ordinal nature of the data, we conducted a sensitivity analysis using the WLSMV estimator, which is specifically designed for ordinal Likert-scale data and uses polychoric correlations. The WLSMV model also demonstrated excellent fit: χ²_robust = 152.847, df = 94, RMSEA = 0.048 (90% CI: 0.039–0.057), CFI = 0.976, and TLI = 0.971. All factor loadings remained statistically significant (*p* < 0.001) and were comparable to those obtained from the ML estimation (ranging from 0.64 to 0.95). The similarity between the ML and WLSMV results confirms that our findings are robust to the choice of estimator and supports the appropriateness of ML estimation with bootstrapping for this dataset.

The one-factor model showed poor fit (χ² = 689.432, df = 104, CFI = 0.812, RMSEA = 0.142, SRMR = 0.118). The two-factor model demonstrated suboptimal fit (χ² = 412.876, df = 103, CFI = 0.902, RMSEA = 0.098, SRMR = 0.087). The higher-order model showed acceptable fit (χ² = 152.654, df = 96, CFI = 0.976, RMSEA = 0.049, SRMR = 0.045). However, the five-factor first-order model exhibited good fit (Δχ² = 12.915, Δdf = 2, *p* < 0.01; AIC = 267.739 vs. 276.654; BIC = 384.162 vs. 396.877), confirming it as the best-fitting and most parsimonious model.

### Convergent and discriminant validity

The SRLS-CNP-P demonstrated strong convergent validity, with all factors achieving CR values between 0.828 and 0.913, all exceeding the recommended threshold of 0.70. Additionally, the AVE ranged from 0.548 to 0.839, with all AVE values surpassing the 0.50 benchmark.

The SRLS-CNP-P demonstrated strong convergent validity, with all factors achieving CR values between 0.828 and 0.913, all exceeding the recommended threshold of 0.70. Additionally, the AVE ranged from 0.548 to 0.839, with all AVE values surpassing the 0.50 benchmark (Table 5). Discriminant validity was confirmed through multiple criteria (Tables 5 and 6). First, according to the Fornell-Larcker criterion, for each factor, the square root of AVE exceeded the correlations between that factor and all other factors (off-diagonal values), confirming that each construct shared more variance with its indicators than with other constructs. Second, for each factor, AVE exceeded both MSV and ASV, further supporting discriminant validity. Third, all HTMT ratios were below the 0.85 threshold, with values ranging from 0.023 to 0.456, and bootstrapped 95% confidence intervals did not contain 1.00 (Table 6). The highest HTMT value was observed between Intrinsic Motivation and Effort Control (0.456; 95% CI: 0.365–0.547), while the lowest was between Achievement Motivation and Multidimensional Thinking (0.023; 95% CI: 0.001–0.052). These findings provide strong evidence that all five factors are empirically distinct despite their theoretical interrelatedness within the self-regulated learning framework.

**Table 5:** Convergent validity and Fornell-Larcker criterion of the SRLS-CNP-P (*n* = 200).

Factors	CR	AVE	MSV	ASV	1	2	3	4	5
1. Intrinsic motivation	0.828	0.548	0.384	0.112	0.74				
2. Achievement motivation	0.886	0.718	0.372	0.096	0.372	0.847			
3. Synthesized knowledge and nursing skills	0.913	0.684	0.372	0.104	0.386	0.104	0.827		
4. Multidimensional thinking	0.912	0.839	0.053	0.021	0.16	0.021	0.053	0.916	
5. Effort control	0.851	0.741	0.384	0.112	0.414	0.372	0.405	0.112	0.861

***Note:*** Diagonal values represent the square root of AVE (√AVE). Off-diagonal values represent factor correlations. For adequate discriminant validity (Fornell-Larcker criterion), each diagonal value should exceed the correlations in its corresponding row and column. **Abbreviations:** CR, Composite Reliability; AVE, Average Variance Extracted; MSV, Maximum Shared Squared Variance; ASV, Average Shared Squared Variance; HTMT, Heterotrait-Monotrait ratio of correlations.

**Table 6:** Heterotrait-Monotrait Ratio (HTMT) and factor correlations of the SRLS-CNP-P (*n* = 200).

Factor Pair	Correlation	HTMT (95% CI)	Interpretation
Intrinsic Motivation ↔ Achievement Motivation	0.372	0.401 (0.317-0.485)	Acceptable
Intrinsic Motivation ↔ Synthesized Knowledge	0.386	0.419 (0.328-0.510)	Acceptable
Intrinsic Motivation ↔ Multidimensional Thinking	0.160	0.178 (0.089-0.267)	Acceptable
Intrinsic Motivation ↔ Effort Control	0.414	0.456 (0.365-0.547)	Acceptable
Achievement Motivation ↔ Synthesized Knowledge	0.104	0.112 (0.048-0.176)	Acceptable
Achievement Motivation ↔ Multidimensional Thinking	0.021	0.023 (0.000-0.052)	Acceptable
Achievement Motivation ↔ Effort Control	0.372	0.413 (0.329-0.497)	Acceptable
Synthesized Knowledge ↔ Multidimensional Thinking	0.053	0.058 (0.012-0.104)	Acceptable
Synthesized Knowledge ↔ Effort Control	0.405	0.443 (0.354-0.532)	Acceptable
Multidimensional Thinking ↔ Effort Control	0.112	0.123 (0.042-0.204)	Acceptable

**Abbreviations:** HTMT, Heterotrait-Monotrait Ratio; CI, Confidence Interval.

### Reliability

The SRLS-CNP-P demonstrated excellent internal consistency, with Cronbach’s alpha (α) ranging from 0.723 to 0.911, McDonald’s omega coefficient ($\omega_{\text{t}}$) between 0.731 and 0.920, Coefficient H values of 0.755–0.931, and Greatest Lower Bound (GLB) estimates of 0.762–0.943 across all factors. All values exceeded 0.70, confirming strong internal consistency. For the total SRLS-CNP-P, reliability coefficients were particularly strong (α = 0.911, $\omega_{\text{t}}\;$= 0.920, H = 0.931, GLB = 0.943), confirming the instrument’s high internal consistency at both factor and SRLS-CNP-P levels. These total-scale coefficients are provided for descriptive purposes only, as the scale is multidimensional and the original developers did not validate a single total score. Interpretation should focus on the validated factor scores and subscale scores.

For the two-item factors, the inter-item correlations were r = 0.721 for Multidimensional Thinking and r = 0.578 for Effort Control, with Spearman-Brown coefficients of 0.838 and 0.733, respectively, indicating acceptable reliability for these brief factors.

Test-retest reliability was assessed in a subsample of 30 nursing students with a two-week interval. The total SRLS-CNP-P demonstrated excellent stability (ICC = 0.872, 95% CI: 0.803–0.947). Although the sample size for test-retest analysis was modest (*n* = 30), all ICC values exceeded the recommended threshold of 0.75, indicating good to excellent reliability. The confidence intervals estimate precision; while some intervals are relatively wide due to the limited sample size (e.g., total scale: 0.803–0.947), the lower bounds of all ICC confidence intervals remain above 0.70, supporting adequate stability (Table 7).

**Table 7:** The results of the reliability of the SRLS-CNP-P.

Factors	Internal consistency (*n* = 200)				Test-retest (*n* = 30)
	α	$\omega_{𝐭}$	H	GLB	ICC (95% CI)
Motivation	0.899	0.902	0.909	0.915	0.837 (0.765-0.884)
Intrinsic motivation	0.821	0.832	0.853	0.877	0.798 (0.702-0.841)
Achievement motivation	0.88	0.891	0.912	0.921	0.783 (0.751-0.831)
Learning strategies	0.867	0.872	0.881	0.889	0.831 (0.774-0.892)
Synthesized knowledge and nursing skills	0.895	0.906	0.927	0.939	0.820 (0.731-0.893)
Multidimensional thinking	0.841	0.853	0.879	0.884	0.804 (0.755-0.892)
Effort control	0.723	0.731	0.755	0.762	0.809 (0.760-0.851)
Total SRLS-CNP-P	0.911	0.92	0.931	0.943	0.872 (0.803-0.947)

***Note:*** Internal consistency estimates (α, $\omega_{\text{t}}$, H, and GLB) were calculated based on the full sample (*n* = 200). Test-retest reliability (ICC) was calculated from a subsample of 30 participants who completed the scale twice, with a two-week interval. **Abbreviations:** α, Cronbach’s alpha; $\omega_{\text{t}}$, McDonald’s omega coefficient; H, Coefficient H; GLB, Greatest Lower Bound; ICC, Intraclass correlation coefficient; CI, Confidence Interval.

## Discussion

This study examined the psychometric properties of the SRLS-CNP-P among Iranian nursing students using a methodology explicitly aligned with COSMIN guidelines for systematic instrument validation [48]. The research evaluates the scale’s validity, reliability, and factor structure through COSMIN-recommended steps: (1) cross-cultural adaptation with linguistic equivalence testing (COSMIN domain 1: Content validity), (2) structural validity via EFA/CFA (Domain 2: Structural validity), and (3) comprehensive reliability testing (Domain 3: Internal consistency and reliability). The findings provide a scientific basis for more accurate evaluation of nursing students’ self-regulated learning abilities in real clinical settings within the study population. Practical applications may include identifying students’ strengths and weaknesses in learning management, designing targeted educational interventions to enhance self-regulation, and utilizing this validated tool in future nursing education research. For educators and administrators, this scale represents a promising instrument for developing professional training programs and improving the quality of clinical nursing education within similar contexts. Future research is needed to confirm its utility for advancing nursing students’ clinical competencies and lifelong learning skills. The study’s outcomes may offer insights for curriculum development and teaching methodologies in similar nursing education programs.

A further consideration concerns the normative framing of self-regulated learning throughout this manuscript. SRL is consistently presented as a desirable attribute associated with academic success, clinical competence, and professional development, which is common in the literature. However, this perspective risks transforming SRL from an analytical construct into a normative ideal. The discussion may imply that improved learning outcomes are primarily a function of individual self-regulation, while contextual factors—such as supervision quality, clinical learning environment characteristics, and institutional support—receive less attention. Research has demonstrated that SRL is shaped by contextual factors, including feedback from clinical educators, the structure of learning opportunities, and the prevailing educational culture [49,50]. A more balanced perspective acknowledges that self-regulation develops through dynamic interaction between individual and environmental influences [9].

Furthermore, several items in the scale (e.g., those emphasizing effort, persistence, independent practice, and achievement) may reflect broader expectations associated with being a “good” nursing student rather than self-regulated learning specifically. High scores could partly capture alignment with professional socialization norms or conformity with educational expectations rather than self-regulated learning alone. This is particularly relevant in clinical education, where students are implicitly and explicitly socialized into professional roles and behaviors [51]. While the scale assesses observable behaviors relevant to clinical learning, users should be aware that scores may reflect both genuine self-regulatory processes and the internalization of professional norms. Future research employing qualitative methods could help disentangle these influences and provide richer insight into what high scores represent in different educational contexts.

Another contextual consideration is the sample’s demographic composition, which was predominantly female (66.7%) and unmarried (86.1%). While this distribution reflects the demographic composition of nursing programs in Iran, where nursing remains a female-dominated profession, the manuscript does not consider whether gendered professional expectations, social roles, or broader cultural factors may shape SRL. Research suggests that gender-specific socialization processes, including expectations around caregiving, responsibility, and academic diligence, can influence self-regulation in educational settings [52,53]. In the Iranian cultural context, where nursing is strongly associated with feminine caregiving roles, female students may experience different motivational pressures and professional socialization compared to their male counterparts [54]. Similarly, marital status may influence students’ available time, social support, and competing responsibilities, potentially affecting their engagement in self-regulatory behaviors. While these demographic factors were not the focus of this validation study, future research should examine whether the SRLS-CNP-P functions equivalently across gender and marital status groups through measurement invariance testing, and explore how social and cultural contexts shape self-regulated learning in clinical nursing education.

An important distinction should be made between linguistic adaptation and cultural equivalence. While our translation procedure ensured semantic, idiomatic, and conceptual equivalence between the English and Persian versions, this does not fully establish that the underlying construct of self-regulated learning is interpreted identically across Japanese, English, and Persian cultural contexts. The original SRLS-CNP was developed in Japan [3], where clinical nursing education emphasizes hierarchical mentor-student relationships, collective learning, and implicit knowledge transmission. In contrast, Iranian nursing education operates within a different cultural framework, characterized by more direct educator-student interactions, explicit performance evaluation, and varying motivational orientations shaped by collectivist values and educational structures. These cultural differences may influence how students interpret items related to achievement motivation (e.g., “getting better grades than classmates”) or synthesized knowledge (e.g., “learning patient materials before practice”). Our findings demonstrate that the scale is understandable and psychometrically stable in the Iranian context; however, true cultural equivalence—the extent to which the construct of SRL carries the same meaning across cultures—remains to be fully established. Future cross-cultural studies employing measurement invariance testing across Japanese, Turkish, and Persian samples would provide stronger evidence of cultural comparability.

In our Persian adaptation, participants confirmed the clarity and relevance of items during face validity assessment, though minor linguistic adjustments were needed to enhance natural phrasing in Persian. The quantitative face validity assessment showed robust Item Impact Scores (2.5–3.2), all exceeding the 1.5 threshold, indicating comprehensibility, as per COSMIN’s emphasis on respondent feedback (Domain 1). Similarly, the Turkish version reported good readability and comprehension in its pilot test, though it did not provide specific quantitative face validity metrics. Both adapted versions showed excellent content validity results, with our Persian version exhibiting I-CVI values of 0.8–1.0 and modified Kappa statistics of 0.79–1.0, while the Turkish version reported CVI values of 0.81–1.0. Notably, while both adaptations retained all original items due to high content validity indices, our Persian version incorporated expert suggestions to refine item wording. In contrast, the Turkish version maintained the items verbatim. These differences likely reflect cultural-linguistic variations between Persian and English compared to Turkish and English, with Persian requiring more nuanced phrasing adjustments to maintain conceptual equivalence while ensuring natural expression. The methodological approach also differed: our study used qualitative feedback and quantitative metrics (including Kappa-adjusted CVIs), providing a more comprehensive validation process than the Turkish version’s [55] reliance on qualitative pilot testing and CVI values alone. These variations do not indicate the superiority of either approach; rather, they show how cultural context and linguistic distance from the original English version influence adaptation strategies. The excellent content validity in both adaptations confirms the fundamental cross-cultural applicability of the scale’s constructs. At the same time, the observed differences in implementation highlight the importance of tailored approaches to instrument adaptation based on target language characteristics and local clinical education contexts. These findings suggest that while the core concepts of self-regulated learning in clinical nursing practice are transferable across cultures, careful attention to linguistic nuances and local educational frameworks remains essential for optimal scale performance in different settings.

Our Persian version demonstrated strong psychometric properties, with exploratory factor analysis extracting five distinct factors with strong loadings (0.75–0.85) accounting for 81.49% of the variance, compared to the Turkish version’s [55] broader range of factor loadings (0.34–0.91). Confirmatory factor analysis showed excellent model fit in our Persian version (CFI = 0.980, RMSEA = 0.044, χ²/df = 1.487), outperforming both the original (CFI = 0.927, RMSEA = 0.066) [3] and Turkish versions (CFI = 0.99, RMSEA = 0.050). The Persian version showed stronger convergent validity (CR = 0.828–0.913; AVE = 0.548–0.839) and was the only version to establish discriminant validity comprehensively across multiple criteria (AVE > MSV/ASV, HTMT < 0.85). These differences in psychometric indices across the three versions should be interpreted with caution, as they may reflect variations in sample characteristics (e.g., age distribution, clinical experience levels), cultural and educational contexts (e.g., different clinical training environments, teaching methodologies), or methodological approaches rather than inherent superiority of any particular adaptation. For instance, the Persian version’s more rigorous cultural-linguistic adaptation process, including cognitive interviews and multiple revisions, may have contributed to stronger factor loadings and a clearer factor structure. Additionally, the application of more advanced psychometric analytical techniques (e.g., multiple reliability indices, comprehensive discriminant validity assessment) in the Persian validation may account for some of the observed differences in reported values. Despite these variations, the overall similarity in factor structure across all three versions confirms the scale’s conceptual robustness across cultures. These findings highlight the importance of considering cultural-linguistic differences and specific clinical education contexts when translating and adapting psychometric instruments.

It should be noted that the discriminant validity established in this study reflects factorial distinctiveness—demonstrating that the five factors are distinguishable from one another within the SRLS-CNP. However, this represents a relatively restricted form of discriminant validity, as all analyses are based on factors derived from the same instrument. The study did not assess whether the SRLS-CNP-P can be distinguished from related but distinct constructs such as self-efficacy, metacognitive regulation, or clinical reasoning ability. Including external measures of related constructs would have provided a more convincing test of construct validity by demonstrating that the scale captures self-regulated learning specifically, rather than overlapping with other theoretically adjacent concepts [56]. Future research should examine discriminant validity against external measures to more fully establish the scale’s construct validity.

An important psychometric observation is the notably high AVE (0.839) for the two-item Multidimensional Thinking factor. While AVE > 0.50 is desirable, values exceeding 0.80 may signal item redundancy [57]. This high AVE likely reflects that the factor has only two items, making the estimate sensitive to inter-item correlations, and that both items (Item 13: developing one’s way of thinking; Item 14: questioning other ways of thinking) share conceptual proximity as they both tap into metacognitive reflection. Nevertheless, the two items are theoretically distinct—Item 13 captures a forward-looking, developmental orientation, while Item 14 reflects a reflective, questioning orientation—consistent with the original framework where multidimensional thinking encompasses both generative and reflective cognitive processes [3]. The original developers also retained this two-item factor, confirming its theoretical importance.

Our Persian version demonstrated strong reliability coefficients, with Cronbach’s alpha ranging from 0.723 to 0.911, McDonald’s omega between 0.731 and 0.920, Coefficient H values of 0.755–0.931, and GLB estimates of 0.762–0.943. These results were comparable to the original version’s [3] reported Cronbach’s alpha of 0.853 for the total scale (with subscales 0.711–0.838) and the Turkish version’s [55] alpha coefficients (total scale 0.898; subscales 0.758–0.871). Test-retest reliability in our Persian version (ICC 0.783–0.872) was consistent with the original version’s correlation of 0.77 and similar to the Turkish version’s [55] 0.878 for the total scale. The observed coefficients in the Persian version were slightly higher in some instances compared to the original and Turkish versions, which may be attributed to several factors. Our cultural adaptation process, including cognitive interviews and multiple pilot tests, may have yielded clearer item phrasing and, consequently, higher internal consistency. The application of advanced reliability measures (omega, Coefficient H, GLB) that better account for scale dimensionality may also explain our more comprehensive estimates compared to studies relying solely on Cronbach’s alpha. However, these differences should not be interpreted as indicating the superiority of any particular version; rather, they likely reflect a combination of methodological, cultural, and sample-related factors that are inherent to cross-cultural validation research.

### Limitations

This study has several limitations that need to be acknowledged. First, the research was conducted at a single medical science university in northern Iran, which may limit the generalizability of the findings to other regions with different educational systems or cultural contexts. Second, although the sample size was adequate for psychometric analyses, participants were limited to undergraduate nursing students, potentially restricting the scale’s applicability to graduate students or practicing nurses. Third, while the two-week test-retest interval was methodologically sound, it may not fully capture the long-term stability of the measured constructs. Additionally, despite rigorous translation procedures, some subtle cultural nuances in the concepts of self-regulated learning may not have been fully captured. The study also did not assess criterion validity against objective clinical performance measures due to practical constraints. Furthermore, the Multidimensional Thinking factor consists of only two items, which demonstrated an unusually high AVE of 0.839. This suggests possible item redundancy or limited construct breadth. While the two items are theoretically distinct, future research should consider adding more items to this subscale to enhance content coverage and potentially improve the AVE to more optimal levels.

Moreover, although the study was designed with reference to the COSMIN standards, it did not assess all COSMIN properties. Measurement error, hypothesis testing against related constructs, responsiveness, and criterion validity were beyond the scope of this study and should be investigated in future research. Lastly, this study assessed convergent validity using the AVE calculated from the CFA model. While this demonstrates convergence of items within each factor, it does not establish convergence with an external measure of self-regulated learning or a theoretically related construct. Therefore, the findings should not be interpreted as establishing external convergent or criterion validity. Future studies should compare the SRLS-CNP-P with other validated measures of self-regulated learning to provide stronger evidence of external validity.

Additionally, although the content validity of the instrument was supported by excellent item-level indices (I-CVI = 0.8–1.0; $K^{*}$ = 0.79–1.0) and a strong S-CVI/Ave of 0.91, the scale-level content validity universal agreement (S-CVI/UA) was 0.75, which falls below the commonly recommended threshold of ≥0.80. This indicates that while most experts rated the items as relevant, agreement was not perfectly unanimous across all items at the scale level. This may be partly attributable to including items that, despite being conceptually relevant, required subtle wording refinements during the qualitative evaluation phase. Nevertheless, this S-CVI/UA value is acknowledged as a limitation, and future studies should consider further item revision or reconvening an expert panel to achieve higher universal agreement; alternatively, they may rely more heavily on the S-CVI/Ave as a more lenient and commonly accepted indicator of content validity for new instruments.

## Conclusion

This study validated the SRLS-CNP-P among undergraduate nursing students at Guilan University of Medical Sciences, demonstrating strong psychometric properties, including excellent reliability, construct validity, and cross-cultural adaptation. The five-factor structure (intrinsic motivation, achievement motivation, synthesized knowledge, multidimensional thinking, and effort control) was confirmed, aligning with the original scale while ensuring cultural and linguistic appropriateness for this population. Furthermore, adherence to COSMIN guidelines and the rigorous translation and validation process enhance confidence in the scale’s applicability in this context. These findings highlight the SRLS-CNP-P as a reliable and valid tool for assessing nursing students’ self-regulated learning behaviors in this setting, offering educators and researchers a means to evaluate and enhance clinical training strategies. However, further validation studies across diverse Persian-speaking populations and educational settings are needed to establish broader generalizability.

Future research should expand on this validation study by examining the scale’s performance across diverse Persian-speaking populations and educational settings to strengthen generalizability. Longitudinal investigations could provide valuable insights into how self-regulated learning skills develop throughout nursing education and their long-term impact on clinical competency. Researchers might also explore the relationship between scale scores and objective clinical performance measures to establish criterion validity. Intervention studies utilizing the SRLS-CNP-P to evaluate educational programs that enhance self-regulated learning could yield important practical applications. Comparative studies with other validated versions of the scale could illuminate cultural influences on self-regulated learning in nursing education. The potential utility of this instrument for assessing practicing nurses’ self-regulated learning behaviors in continuing education contexts also warrants exploration. Finally, qualitative studies could complement these quantitative findings by better understanding the contextual factors influencing self-regulated learning in clinical nursing practice. These future directions would collectively help optimize nursing education and clinical training by improving knowledge and measurement of self-regulated learning processes.

## Supporting information

S1 File English version of the SRLS-CNP. (DOCX)

S2 File Back translation of the SRLS-CNP. (DOCX)

S3 File Data. (XLSX)

S4 File Table S1. (DOCX)

## References

[1] Higgins N, Frankland S, Rathner J. Self-regulated learning in undergraduate science. IJISME. 2021;29(1). doi: 10.30722/ijisme.29.01.005

[2] Baluwa MA, Lazaro M, Mhango L, Msiska G. Stress and coping strategies among Malawian undergraduate nursing students. Adv Med Educ Pract. 2021;12:547–56. doi: 10.2147/AMEP.S300457 34093050 PMC8169817

[3] Iyama S, Maeda H. Development of the self-regulated learning scale in clinical nursing practice for nursing students: Consideration of its reliability and validity. Jpn J Nurs Sci. 2018;15(3):226–36. doi: 10.1111/jjns.12191 29152853

[4] Ozsaker E, Aykut Z, Doruker NC, Koze BS, Gecit S. The relationship between the academic self-efficacy and perceived stressors among nursing students in clinical settings: A cross-sectional study. BMC Nurs. 2025;24(1):216. doi: 10.1186/s12912-025-02836-0 40011931 PMC11863915

[5] Mohammadi F, Hosseini SK, Khazaei S, Fariba F. Psychometrics assessment of clinical reasoning competency scale in nurses of critical care units. BMC Nurs. 2024;23(1):733. doi: 10.1186/s12912-024-02263-7 39385139 PMC11465576

[6] Albagawi BS, Alsalamah YS, Nashwan AJ, Rawili RMA, Babkair LA, Alkharji SA, et al. The mediating role of learning motivation in the relationship among perceived stress and emotional regulation among Saudi nursing students in clinical practice. BMC Nurs. 2024;23(1):245. doi: 10.1186/s12912-024-01893-1 38627769 PMC11020197

[7] Sadeghi N, Janatolamakn M, Rezaeian S, Rashi M, Khatony A. Exploring self-directed learning readiness and related factors: The role of time management skills in nursing students. BMC Med Educ. 2024;24(1):1088. doi: 10.1186/s12909-024-06083-w 39363354 PMC11450985

[8] Aliakbari F, Parvin N, Heidari M, Haghani F. Learning theories application in nursing education. J Educ Health Promot. 2015;4:2. doi: 10.4103/2277-9531.151867 25767813 PMC4355834

[9] Zimmerman BJ. Becoming a self-regulated learner: An overview. Theory Pract. 2002;41(2):64–70. doi: 10.1207/s15430421tip4102_2

[10] Pintrich PR. A conceptual framework for assessing motivation and self-regulated learning in college students. Educ Psychol Rev. 2004;16(4):385–407. doi: 10.1007/s10648-004-0006-x

[11] Panadero E. A review of self-regulated learning: Six models and four directions for research. Front Psychol. 2017;8:422. doi: 10.3389/fpsyg.2017.00422 28503157 PMC5408091

[12] Schunk DH, Zimmerman B. Handbook of self-regulation of learning and performance. Taylor & Francis; 2011.

[13] Cho S, Jang SJ. Nursing students’ motivational and self-regulated learning during the COVID-19 pandemic: A cross-sectional study. Nurs Health Sci. 2022;24(3):699–707. doi: 10.1111/nhs.12968 35717611 PMC9349992

[14] Tolyat M, Abolfazl Vagharseyyedin S, Nakhaei M. Education of nursing profession amid COVID-19 Pandemic: A qualitative study. J Adv Med Educ Prof. 2022;10(1):39–47. doi: 10.30476/JAMP.2021.90779.1422 34981004 PMC8720149

[15] Kaveh O, Charati FG, Kamali M, Mojarrad FA. Clinical nursing education during the COVID-19 pandemic: Perspectives of students and clinical educators. BMC Nurs. 2022;21(1):286. doi: 10.1186/s12912-022-01029-3 36289535 PMC9598001

[16] Gonzalez-Garcia A, Bermejo-Martinez D, Lopez-Alonso AI, Trevisson-Redondo B, Martín-Vázquez C, Perez-Gonzalez S. Impact of ChatGPT usage on nursing students education: A cross-sectional study. Heliyon. 2024;11(1):e41559. doi: 10.1016/j.heliyon.2024.e41559 39850430 PMC11755058

[17] Jonathan S. Nursing in the digital age: The importance of health technology and its advancement in nursing and healthcare. In: Digital technology in public health and rehabilitation care. Elsevier; 2025. p. 283–96. doi: 10.1016/b978-0-443-22270-2.00018-6

[18] van Hooft S, Berger E, van Torenburg C, van Staa A. Daily routines, short-term priorities, and nurses’ role hamper self-management support in a hospital setting: A mixed methods study. Int J Nurs Stud Adv. 2024;8:100279. doi: 10.1016/j.ijnsa.2024.100279 39720108 PMC11667052

[19] Keçeci A. Self-regulated learning in nursing: A study from a health education course. J Hum Sci. 2017;14(4):3830. doi: 10.14687/jhs.v14i4.4415

[20] Sandars J, Cleary TJ. Self-regulation theory: Applications to medical education: AMEE Guide No. 58. Med Teach. 2011;33(11):875–86. 10.3109/0142159X.2011.595434 22022899

[21] Kurt E, Eskimez Z. Examining self-regulated learning of nursing students in clinical practice: A descriptive and cross-sectional study. Nurse Educ Today. 2022;109:105242. doi: 10.1016/j.nedt.2021.105242 34922139

[22] Peck B, Smith A, Terry D, Porter JE. Self-regulation for and of learning: Student insights for online success in a Bachelor of nursing program in regional Australia. Nurs Rep. 2021;11(2):364–72. doi: 10.3390/nursrep11020035 34968213 PMC8608067

[23] Tomas N, Poroto A. The interplay between self-regulation, learning flow, academic stress and learning engagement as predictors for academic performance in a blended learning environment: A cross-sectional survey. Heliyon. 2023;9(11):e21321. doi: 10.1016/j.heliyon.2023.e21321 37885718 PMC10598534

[24] An J, Oh J, Park K. Self-regulated learning strategies for nursing students: A pilot randomized controlled trial. Int J Environ Res Public Health. 2022;19(15):9058. doi: 10.3390/ijerph19159058 35897439 PMC9331953

[25] Mokkink LB, Terwee CB, de Vet HC. COSMIN: Consensus-based standards for the selection of health status measurement instruments. In: Encyclopedia of quality of life and well-being research. Springer. 2024. p. 1445–8.

[26] Wolf EJ, Harrington KM, Clark SL, Miller MW. Sample size requirements for structural equation models: An evaluation of power, bias, and solution propriety. Educ Psychol Meas. 2013;76(6):913–34. doi: 10.1177/0013164413495237 25705052 PMC4334479

[27] Kline RB. Principles and practice of structural equation modeling. Guilford publications; 2023.

[28] Goodboy AK, Kline RB. Statistical and practical concerns with published communication research featuring structural equation modeling. Commun Res Rep. 2016;34(1):68–77. doi: 10.1080/08824096.2016.1214121

[29] dos Santos PM, Cirillo MÂ. Construction of the average variance extracted index for construct validation in structural equation models with adaptive regressions. Commun Stat - Simul Comput. 2021;52(4):1639–50. doi: 10.1080/03610918.2021.1888122

[30] Sarstedt M, Ringle CM, Hair JF. Partial least squares structural equation modeling. In: Handbook of market research. Springer; 2021. p. 587–632.

[31] Bornstein RF. Face validity in psychological assessment: Implications for a unified model of validity. 1996.

[32] Polit DF, Beck CT. The content validity index: Are you sure you know what’s being reported? Critique and recommendations. Res Nurs Health. 2006;29(5):489–97. doi: 10.1002/nur.20147 16977646

[33] Polit DF, Beck CT, Owen SV. Is the CVI an acceptable indicator of content validity? Appraisal and recommendations. Res Nurs Health. 2007;30(4):459–67. doi: 10.1002/nur.20199 17654487

[34] Pett MA, Lackey NR, Sullivan JJ. Making sense of factor analysis: The use of factor analysis for instrument development in health care research. Sage; 2003.

[35] George D, Mallery P. IBM SPSS statistics 29 step by step: A simple guide and reference. Routledge; 2024.

[36] Byrne BM. Structural equation modeling with Mplus: Basic concepts, applications, and programming. Routledge; 2013.

[37] Muthen L, Muthen B. Mplus statistical analysis with latent variables: user’s guide. Version. 8th ed. Los Angeles (CA): Muthén & Muthén; 2017.

[38] Finney SJ, DiStefano C. Nonnormal and categorical data in structural equation modeling. 2013.

[39] S S, Mohanasundaram T. Fit indices in structural equation modeling and confirmatory factor analysis: Reporting guidelines. Asian J Econ Busin Acc. 2024;24(7):561–77. doi: 10.9734/ajeba/2024/v24i71430

[40] Fornell C, Larcker DF. Evaluating structural equation models with unobservable variables and measurement error. J Mark Res. 1981;18(1):39. doi: 10.2307/3151312

[41] Henseler J, Ringle CM, Sarstedt M. A new criterion for assessing discriminant validity in variance-based structural equation modeling. J Acad Mark Sci. 2014;43(1):115–35. doi: 10.1007/s11747-014-0403-8

[42] Malkewitz CP, Schwall P, Meesters C, Hardt J. Estimating reliability: A comparison of Cronbach’s α, McDonald’s ωt and the greatest lower bound. Soc Sci Humanit Open. 2023;7(1):100368. doi: 10.1016/j.ssaho.2022.100368

[43] McNeish D. Thanks coefficient alpha, we’ll take it from here. Psychol Methods. 2018;23(3):412–33. doi: 10.1037/met0000144 28557467

[44] Kalkbrenner MT. Alpha, omega, and H internal consistency reliability estimates: Reviewing these options and when to use them. Couns Outcome Res Eval. 2021;14(1):77–88. doi: 10.1080/21501378.2021.1940118

[45] Eisinga R, Grotenhuis M, Pelzer B. The reliability of a two-item scale: Pearson, Cronbach, or Spearman-Brown? Int J Public Health. 2013;58(4):637–42. doi: 10.1007/s00038-012-0416-3 23089674

[46] Taber KS. The use of Cronbach’s alpha when developing and reporting research instruments in science education. Res Sci Educ. 2017;48(6):1273–96. doi: 10.1007/s11165-016-9602-2

[47] Koo TK, Li MY. A guideline of selecting and reporting intraclass correlation coefficients for reliability research. J Chiropr Med. 2016;15(2):155–63. doi: 10.1016/j.jcm.2016.02.012 27330520 PMC4913118

[48] Mokkink LB, Terwee CB, Patrick DL, Alonso J, Stratford PW, Knol DL, et al. The COSMIN checklist for assessing the methodological quality of studies on measurement properties of health status measurement instruments: an international Delphi study. Qual Life Res. 2010;19(4):539–49. doi: 10.1007/s11136-010-9606-8 20169472 PMC2852520

[49] Pintrich PR, Zusho A. Student motivation and self-regulated learning in the college classroom. In: The scholarship of teaching and learning in higher education: An evidence-based perspective. Springer; 2007. p. 731–810.

[50] Boekaerts M, Corno L. Self‐regulation in the classroom: A perspective on assessment and intervention. Appl Psychol. 2005;54(2):199–231. doi: 10.1111/j.1464-0597.2005.00205.x

[51] Benner P, Sutphen M, Leonard V, Day L. Educating nurses: A call for radical transformation. John Wiley & Sons; 2009.

[52] Kitsantas A, Winsler A, Huie F. Self-regulation and ability predictors of academic success during college: A predictive validity study. J Adv Acad. 2008;20(1):42–68. doi: 10.4219/jaa-2008-867

[53] Matthews JS, Ponitz CC, Morrison FJ. Early gender differences in self-regulation and academic achievement. J Educ Psychol. 2009;101(3):689–704. doi: 10.1037/a0014240

[54] Hakami R. Men studying to be nurses: An exploration of the lived experience of men in undergraduate nursing education. University of British Columbia; 2020.

[55] Baysan A, Orgun F. Psychometric properties of the Turkish version of the self-regulated learning scale in clinical nursing practice. J Educ Res Nurs. 2023;:374–9. doi: 10.14744/jern.2023.22533

[56] Campbell DT, Fiske DW. Convergent and discriminant validation by the multitrait-multimethod matrix. Psychol Bull. 1959;56(2):81–105. doi: 10.1037/h0046016 13634291

[57] Hair JF, Black WC, Babin BJ, Anderson RE, Black W, Anderson R. Multivariate data analysis, vol. 4. 8th ed. Cengage Learning EMEA; 2019. p. 95–120.

