# Supplementary material for: Psychometric properties of the Persian version of the self-regulated learning scale in clinical nursing practice for nursing students: A methodological study
Source: PLoS One. 2026 Sep 25;21(9):e0358790. doi: 10.1371/journal.pone.0358790 (PMC13614530; doi:10.1371/journal.pone.0358790)
Supplement: Supplementary file 1 [file pone.0358790.s005.pdf]

PLOS ONE Publication Criteria Assessment

Overview of PLOS ONE's Publication Criteria

PLOS ONE evaluates manuscripts based on scientific validity, methodological rigor, and ethical standards, not on perceived importance or novelty of findings. To be accepted, research articles must satisfy seven specific criteria:

Criterion 1: The study presents the results of original research.

- The study reports a psychometric validation of the Self-Regulated Learning Scale in Clinical Nursing Practice (SRLS-CNP) adapted for Persian-speaking nursing students. Methodological studies validating existing instruments for new cultural contexts constitute original research within PLOS ONE's scope.
- The scale was originally developed by Iyama and Maeda (2018) in Japan. This study reports on cross-cultural adaptation and validation in Iran—constituting new research contributions regarding the instrument's performance in a novel linguistic and cultural context. No prior Persian validation of this scale exists (the manuscript itself states "in the Iranian context, the lack of culturally relevant assessment tools hinders our understanding of students' self-regulatory behaviors").
-

  Main Claims of the Paper:
- The authors claim that the translated instrument demonstrates strong content validity, construct validity, convergent and discriminant validity, internal consistency, and test–retest reliability, making it an appropriate tool for assessing self-regulated learning in Persian-speaking nursing students. The study addresses an important methodological gap, as culturally appropriate measurement tools are essential for advancing educational research and evaluating interventions within specific linguistic and cultural contexts. Although instrument validation studies are inherently incremental rather than conceptually novel, the manuscript makes a worthwhile contribution to nursing education by providing an evidence-based assessment tool for future research and educational practice.

Criterion 2: Results reported have not been published elsewhere.

- The authors state the study is original and no prior Persian validation exists. The manuscript compares findings to the original Japanese version and a Turkish adaptation published in 2023 [citation:41]. No evidence of prior publication.

Criterion 3: Experiments, statistics, and analyses are performed to a high technical standard and described in sufficient detail.

- Assessment: Manuscript partially meets criteria—requires clarification
- Strengths:
- COSMIN guideline adherence: The study explicitly follows COSMIN (Consensus-based Standards for the selection of health Measurement Instruments) checklists, an internationally recognized standard for instrument validation [citation:40].
- Comprehensive psychometric testing: Face validity (qualitative and quantitative), content validity (I-CVI, K*), construct validity (EFA with promax rotation, CFA with multiple fit indices), convergent validity (CR, AVE), discriminant validity (Fornell-Larcker, MSV/ASV, HTMT), and reliability (Cronbach's α, McDonald's ω, Coefficient H, GLB, ICC).
- Appropriate sample size: EFA used 160 participants (10:1 ratio), CFA used 200 participants (exceeding minimum threshold of 200 recommended) [citation:25, 26].
- Statistical detail: Adequate reporting of factor loadings, eigenvalues, fit indices, and thresholds.
- Methodological Issues Requiring Clarification:
- Issue Concern Recommendation
- Estimation method
  Maximum likelihood estimation used for both EFA and CFA despite five-point ordinal response categories; no assessment of multivariate normality or justification provided
  Justify use of ML with ordinal data; consider robust estimation methods (e.g., MLR) or report normality assessment
- Factor retention
  Authors report retention based on eigenvalues, scree plots, and parallel analysis, but parallel analysis and scree plot results are not presented
  Include scree plot figure and parallel analysis results in manuscript or supplementary materials
- Model modifications
  Manuscript does not specify whether modification indices, correlated residuals, or other post hoc model adjustments were used
- Clarify if any model modifications were made; report any modifications transparently
- RMSEA reporting
- RMSEA 90% CI is mentioned but not provided

- Include RMSEA 90% CI for full transparency
- Missing data
- No information provided on handling of missing data
- Report if missing data occurred and how it was addressed
- Additional Considerations:
- The manuscript states participants were "distributed across academic years as follows: second year (33.3%), third year (30.6%), fourth year (36.1%)"—a total of 100%, and the numbers given (120+110+130=360) sum correctly.
- Single-site design (only Guilan University of Medical Sciences), which the authors acknowledge as a limitation. This is not fatal to methodological rigor but affects generalizability.
- The manuscript should confirm that independent samples were used for EFA and CFA.

Criterion 4: Conclusions are presented in an appropriate fashion and are supported by the data.

- Assessment: Partially meets criteria—interpretation requires caution
- Are the claims properly placed in the context of the previous literature?
- The manuscript is generally well situated within the existing literature. The authors provide an appropriate overview of self-regulated learning theory, its relevance to nursing education, and the development of the original Japanese instrument. They also compare their findings with those reported for both the original and Turkish versions of the scale.
- Concerns Regarding Interpretation:
- Parts of the discussion overstate the comparative performance of the Persian version. Statements suggesting that the present version demonstrates "superior" psychometric properties should be interpreted more cautiously. Differences in model fit and reliability may reflect differences in sample characteristics, cultural contexts, and analytical approaches rather than true superiority of the instrument. A more balanced interpretation acknowledging these factors would strengthen the discussion and more accurately reflect the available evidence.
- The conclusions are generally proportionate regarding the primary findings, but the discussion contains considerable repetition of results already presented in the Results section. The repeated use of terms such as "excellent," "robust," "strong," and "superior" results in an unnecessarily promotional tone and should be replaced with more academically appropriate language. Greater emphasis on interpreting the practical implications of the findings, rather than reiterating statistical outcomes, would improve readability and scientific objectivity.

- COSMIN Compliance Clarification:
- The authors state that the study followed COSMIN guidelines, and the manuscript generally adheres to accepted practices for psychometric validation studies. However, the discussion occasionally implies complete adherence to COSMIN despite several measurement properties—including criterion validity, responsiveness, measurement error, and measurement invariance—not being assessed. The authors should clarify that the study addresses the core measurement properties relevant to this stage of instrument validation rather than implying comprehensive COSMIN compliance.

Criterion 5: The article is presented in an intelligible fashion and is written in standard English.

- Assessment: Meets criteria (with minor issues)
- The manuscript is generally well-organised and written in clear English. The methods are logically presented, and the overall structure is easy to follow. However:
- Minor formatting errors: duplicated table entries for items 3, 4, 5 show "Excellence" instead of "Excellent" in the content validity table.
- Some minor phrasing could be improved for clarity.
- The discussion is longer than necessary and contains considerable repetition of the results. The focus should be on clarification of what the results mean
- Some longer sentences could be simplified to improve readability for a broader readership.

PLOS ONE notes that it does not copyedit accepted manuscripts, so authors should correct any errors at revision.

Criterion 6: The research meets all applicable standards for the ethics of experimentation and research integrity.

- Assessment: Meets criteria
- Ethical approval: Explicitly stated—IR.GUMS.REC.1403.040 from Guilan University of Medical Sciences Research Ethics Committee.
- Helsinki Declaration compliance: Stated.
- Informed consent: Written informed consent obtained from all participants.
- Participant rights: Voluntary participation, freedom to withdraw, confidentiality ensured.

  Dual Use Research:

- No. This research does not involve any experiments or methodologies that would be considered dual-use research of concern under current NIH definitions.

Criterion 7: The article adheres to appropriate reporting guidelines and community standards for data availability.

- Assessment: Partially meets criteria—significant concern
- Reporting Guidelines:
- The study explicitly follows COSMIN guidelines, appropriate for a validation study.
- The manuscript includes adequate reporting of methodological details (translation process, validity and reliability assessment).
- Data Availability—MAJOR CONCERN:
- Current Data Availability Statement:
- "The datasets used and analyzed during this study are available from the corresponding author upon reasonable request."
- This DOES NOT meet PLOS ONE requirements. PLOS ONE's data availability policy requires authors to make all data underlying the findings fully available without restriction, with rare exception. The policy explicitly states:
- "The PLOS Data policy requires authors to make all data underlying the findings described in their manuscript fully available without restriction... The data should be provided as part of the manuscript or its supporting information, or deposited to a public repository."
-
- "Available upon reasonable request" is not acceptable under PLOS ONE policy. Possible solutions:
- Deposit de-identified data in a public repository (e.g., Figshare, Dryad, OSF, Zenodo)
- Provide data as Supporting Information files
- If ethical restrictions apply (participant privacy concerns), these must be detailed and justified—but "available from corresponding author" alone is not acceptable.
- Additional Journal Requirements
- Requirement Status
- Competing Interests Authors declare none.
- Financial Disclosure "The author(s) received no specific funding for this work."
- Publication Fees
- No assessment possible from reviewer materials.
- License
- Authors should be prepared to sign CC BY license.
- Software and Reproducibility Assessment

- Are details of the methodology sufficient to allow the experiments to be reproduced?
- For the most part, yes. The sampling procedures, translation process, validity assessments, reliability analyses, and statistical methods are described in sufficient detail for replication. However, reproducibility would be improved through:
- Providing the complete translation protocol and expert review documentation as supplementary material
- Publishing the scoring procedures
- Depositing statistical syntax (SPSS and AMOS) as supplementary material
- Additional reporting of:
- Factor retention process (scree plot, parallel analysis results)
- Assessment of statistical assumptions
- Handling of missing data
- Details regarding any CFA model modifications
-
- Is any software created by the authors freely available?
  Not applicable.
-
- Overall Recommendation
  Decision: Major Revision
-
- Justification:
  The study is methodologically sound and addresses a meaningful gap in nursing education research. The psychometric validation is rigorous, following COSMIN guidelines with appropriate statistical analyses. However, several issues require attention before acceptance:
-
- Summary of Required Changes:
- Critical Issues (Must Address):
- Data Availability Statement: Revise to comply with PLOS ONE policy—either:
- Deposit data in a public repository and provide a DOI/URL
- Upload data as Supporting Information
- Provide detailed justification for any restrictions (if applicable)
- Methodological Clarifications Required:
- Justify maximum likelihood estimation with ordinal data or report normality assessment
- Present parallel analysis and scree plot results
- Clarify if any CFA model modifications were made (including modification indices or correlated residuals)
- Provide RMSEA 90% CI

- Report handling of missing data
- Confirm independent samples used for EFA and CFA
- Interpretation Revisions:
- Replace promotional language ("excellent," "robust," "strong," "superior") with more academically appropriate terms
- Avoid suggesting "superiority" of Persian version over previous adaptations—differences may reflect sample/cultural variations
- Clarify that the study addresses core measurement properties rather than implying complete COSMIN compliance
- Minor Issues (Should Address):
- Correct typographical errors (e.g., "Excellence" → "Excellent" in content validity table)
- Light language copyediting:
- Shorten the discussion to avoid repetition
- Simplify longer sentences where possible
- Consider providing supplementary materials:
- Complete translation protocol
- Expert review documentation
- Statistical analysis syntax (SPSS and AMOS)

- Recommendation for Editors:
- This manuscript is scientifically valid and would likely be publishable in PLOS ONE after the authors address the critical issues identified. No fatal methodological or ethical flaws were identified. The concerns relate primarily to data availability, statistical reporting transparency, interpretation of findings, and language rather than fundamental flaws in study design.
-
