# Supplementary material for: Psychometric properties of the Persian version of the self-regulated learning scale in clinical nursing practice for nursing students: A methodological study
Source: PLoS One. 2026 Sep 25;21(9):e0358790. doi: 10.1371/journal.pone.0358790 (PMC13614530; doi:10.1371/journal.pone.0358790)
Supplement: Supplementary file 2 [file pone.0358790.s001.docx]

**English version of the SRLS-CNP**

| **Item** | **Strongly Disagree** | **Disagree** | **Neutral** | **Agree** | **Strongly Agree** |
| --- | --- | --- | --- | --- | --- |
| 1. I like clinical nursing practice even though it is difficult. |  |  |  |  |  |
| 2. I like clinical nursing practice even if I cannot get good grades. |  |  |  |  |  |
| 3. I like clinical nursing practice because I can learn new things. |  |  |  |  |  |
| 4. I am satisfied with myself because I am working hard during clinical nursing practice. |  |  |  |  |  |
| 5. It is important for me to get good grades in clinical nursing practice. |  |  |  |  |  |
| 6. I want to get better grades than my classmates for clinical nursing practice. |  |  |  |  |  |
| 7. I would like to demonstrate my ability to others by getting good grades in clinical nursing practice. |  |  |  |  |  |
| 8. I confirm clinical nursing skills before providing care to patients. |  |  |  |  |  |
| 9. Using my own words, I want to explain important points I have learned in my clinical nursing practice. |  |  |  |  |  |
| 10. I think about ways to implement care tailored to individual patient needs. |  |  |  |  |  |
| 11. I learn patient materials before clinical nursing practice. |  |  |  |  |  |
| 12. I practice nursing skills by myself during a clinical nursing practice period. |  |  |  |  |  |
| 13. I would like to develop my way of thinking based on what I have learned through clinical nursing practice. |  |  |  |  |  |
| 14. I wonder if there are other ways of thinking about what I have learned through clinical nursing practice. |  |  |  |  |  |
| 15. I make an effort to understand difficult concepts I have learned from clinical mentors and instructors. |  |  |  |  |  |
| 16. I try to associate what I have experienced through clinical nursing practice with the knowledge I previously acquired. |  |  |  |  |  |
