# Supplementary material for: Psychometric properties of the Persian version of the self-regulated learning scale in clinical nursing practice for nursing students: A methodological study
Source: PLoS One. 2026 Sep 25;21(9):e0358790. doi: 10.1371/journal.pone.0358790 (PMC13614530; doi:10.1371/journal.pone.0358790)
Supplement: Supplementary file 3 [file pone.0358790.s002.docx]

**Back translation of the Self-Regulated Learning Scale in Clinical Nursing Practice for nursing students (in Persian)**

Motivation subscale

1. I enjoy engaging in clinical nursing practice despite its challenges.

2. I enjoy working in clinical nursing despite not achieving high grades.

3. I enjoy engaging in clinical nursing practice because it allows me to acquire new knowledge.

4. I am content with my efforts as I dedicate myself to clinical nursing practice.

5. Getting good grades in clinical nursing practice is crucial to me.

6. I aim to achieve higher grades than my peers in my clinical nursing practice.

7. I want to showcase my skills to others by achieving high scores in clinical nursing practice.

Learning strategies subscale

8. Before providing care to patients, I ensure I have verified my clinical nursing skills.

9. I want to elucidate key concepts that I've gained from my experience in clinical nursing, using my own language.

10. I consider methods for customizing care based on each patient's unique needs.

11. I study patient materials before engaging in clinical nursing practice.

12. I hone my nursing skills independently while completing a clinical nursing practice period.

13. I want to enhance my critical thinking skills through the knowledge gained from my experience in clinical nursing practice.

14. I am curious if alternative perspectives exist for considering the knowledge gained from my experience in clinical nursing.

15. I strive to comprehend challenging concepts that clinical mentors and instructors have taught me.

16. The knowledge I gained before is something I seek to connect with my experiences in clinical nursing practice.
