# Supplementary material for: Psychometric properties of the Persian version of the self-regulated learning scale in clinical nursing practice for nursing students: A methodological study
Source: PLoS One. 2026 Sep 25;21(9):e0358790. doi: 10.1371/journal.pone.0358790 (PMC13614530; doi:10.1371/journal.pone.0358790)
Supplement: Supplementary file 4 [file pone.0358790.s004.docx]

**Table S1.** Item-level descriptive statistics and floor/ceiling effects of the SRLS-CNP-P (*n* = 360)

| **Items** | **Mean** | **SD** | **Skewness** | **Kurtosis** | **Floor (%)** | **Ceiling (%)** |
| --- | --- | --- | --- | --- | --- | --- |
| 1 | 3.6 | 0.71 | -0.42 | 0.18 | 1.4% | 8.3% |
| 2 | 3.68 | 0.74 | -0.51 | 0.09 | 1.1% | 9.7% |
| 3 | 3.81 | 0.73 | -0.58 | 0.22 | 0.8% | 11.4% |
| 4 | 3.59 | 0.72 | -0.38 | -0.11 | 1.7% | 7.8% |
| 5 | 3.72 | 0.73 | -0.55 | 0.14 | 1.4% | 10.3% |
| 6 | 3.75 | 0.56 | -0.31 | -0.22 | 0.6% | 8.9% |
| 7 | 3.08 | 0.51 | -0.08 | -0.45 | 2.2% | 4.2% |
| 8 | 3.74 | 0.48 | -0.47 | 0.31 | 0.3% | 9.4% |
| 9 | 2.42 | 0.75 | 0.33 | -0.52 | 8.6% | 1.9% |
| 10 | 3.72 | 0.43 | -0.44 | 0.42 | 0.3% | 8.6% |
| 11 | 3.45 | 0.62 | -0.28 | -0.08 | 1.9% | 6.1% |
| 12 | 3.52 | 0.58 | -0.32 | -0.15 | 1.4% | 6.7% |
| 13 | 3.61 | 0.55 | -0.28 | -0.19 | 1.1% | 6.9% |
| 14 | 3.48 | 0.59 | -0.21 | -0.33 | 1.7% | 5.8% |
| 15 | 3.57 | 0.61 | -0.33 | -0.12 | 1.4% | 7.5% |
| 16 | 3.62 | 0.57 | -0.38 | -0.05 | 0.8% | 7.8% |

**Abbreviations:** SD, Standard Deviation; Floor %, Percentage of respondents selecting score 1; Ceiling %, Percentage of respondents selecting score 5.
