# Supplementary material for: Psychometric properties of the Persian version of the self-regulated learning scale in clinical nursing practice for nursing students: A methodological study
Source: PLoS One. 2026 Sep 25;21(9):e0358790. doi: 10.1371/journal.pone.0358790 (PMC13614530; doi:10.1371/journal.pone.0358790)
Supplement: Supplementary file 5 [file pone.0358790.s007.docx]

***PLOS One***

**Academic Editor**

**Dear Professor De-Chih Lee,**

Thank you for considering the revised version of our manuscript entitled "**Psychometric properties of the Persian version of the self-regulated learning scale in clinical nursing practice for nursing students: A methodological study**." We appreciate the editor and reviewers for highlighting essential modifications required for the manuscript. Their insightful comments have been incredibly valuable in restructuring our work. We have carefully taken these suggestions into account. A detailed explanation of the changes made in response to the reviewers' feedback is provided point by point in the following pages, with all modifications highlighted in yellow within the manuscript. We sincerely hope that these revisions meet the criteria for making the manuscript acceptable for publication in *PLOS One*.

We are looking forward to hearing from you.

| **Page(s)** | **Author response to comments** | **Comments** |
| --- | --- | --- |
|  | **Reviewer 1** | |
| 5-6 | We would like to express sincere gratitude for dedicating your valuable time to review our manuscript. Your insightful comments and detailed feedback have been instrumental in improving the overall quality and clarity of our work.  In response to your valuable suggestion, we have revised the manuscript to explicitly describe the random division process.  We are grateful for your time and expertise in reviewing our work. | 1. The sample size justification is adequate for the analyses performed, but the handling of the split‑sample design (EFA n=160, CFA n=200) is not clearly tied to the original sample size calculation. The authors state that they aimed for 360 participants to meet both EFA and CFA requirements, which is appropriate. However, they do not report how the 360 were randomly divided into the two subsamples. Random splitting is acceptable, but the method should be described (e.g., using a random number generator in SPSS). Without this description, the independence of the EFA and CFA samples cannot be fully verified. |
| 14-15 | We sincerely thank you for your insightful and valuable comment regarding the reporting of parallel analysis results. Your attention to this methodological detail is greatly appreciated.  In response to your suggestion, we have revised the Results section to include the complete parallel analysis output. Specifically, we now report the actual eigenvalues alongside the 95th percentile random eigenvalues for all factors, demonstrating that the first five factors exceeded the random thresholds while the sixth did not. This confirms the five-factor solution was supported across all three retention criteria.  We are deeply grateful for your time and expertise in reviewing our manuscript. | 2. The exploratory factor analysis used maximum likelihood extraction with Promax rotation, which is reasonable given the expected correlation among factors. However, the authors report using multiple criteria for factor retention (eigenvalue >1, scree plot, parallel analysis), but in the results section only eigenvalues and cumulative variance are presented. The parallel analysis results are not reported. Parallel analysis is generally more accurate than the eigenvalue >1 rule, so its omission is a notable gap. The authors should either provide the parallel analysis output (e.g., comparing real‑data eigenvalues to random‑data eigenvalues) or state clearly that parallel analysis was not performed despite being mentioned in the methods. If it was performed, the results must be reported. |
| 16-17 and Table 4 | We truly appreciate your thorough and constructive feedback regarding the reporting of our CFA results. Your suggestion to improve the transparency of our factor loading reporting is highly valued.  In response to your valuable comment, we have added a comprehensive table (Table 4) presenting all standardized factor loadings with their corresponding standard errors, critical ratios, and p-values for each of the 16 items, organized by their respective latent factors. This allows readers to easily identify which items have lower loadings (e.g., Item 12 with a standardized loading of 0.66) and verify the magnitude of all loadings. Additionally, we have clarified in the text that the critical ratios are derived from unstandardized estimates and standard errors, and we have noted that Figure 1 is provided as a separate file per PLOS ONE submission guidelines.  We are sincerely grateful for the time and care you have dedicated to reviewing our manuscript. Your expertise has been invaluable in strengthening this work. | 3. The confirmatory factor analysis model fit is excellent, but the authors do not report the standardized factor loadings from the CFA model in the text or in a table. They provide a path diagram (Figure 1) that is not visible in the review file, so the reader cannot verify the magnitude of the loadings. The text only states that loadings ranged from 0.66 to 0.96. This range should be presented with item numbers to allow assessment of which items have lower loadings. Additionally, the critical ratios (C.R.) are reported for a few items (e.g., “from 5.698 to 21.485”), but these are derived from unstandardized estimates and standard errors. Reporting the standardized loadings with their standard errors or t‑values would be more informative. |
| 24 and 26 | We sincerely thank you for this insightful and methodologically important comment regarding the high AVE for the Multidimensional Thinking factor. Your careful observation is greatly appreciated and has helped us provide a more balanced interpretation of our findings.  In response to your valuable suggestion, we have added a dedicated paragraph in the Discussion section acknowledging this issue. We explain that the high AVE (0.839) likely reflects that the factor consists of only two items, making the estimate sensitive to inter-item correlations, and that both items share conceptual proximity as they tap into metacognitive reflection. We also emphasize that despite the statistical concern, the two items are theoretically distinct—Item 13 captures a forward-looking, developmental orientation, while Item 14 reflects a reflective, questioning orientation—consistent with the original framework. We have also acknowledged this as a limitation and recommended future research to expand this subscale with additional items.  We are deeply grateful for your time, expertise, and the thoughtful attention you have given to our manuscript. | 4. The convergent validity results are strong, but the average variance extracted (AVE) for the multidimensional thinking factor is 0.839, which is very high. An AVE above 0.80 can sometimes indicate item redundancy, especially given that this factor has only two items. The authors should comment on whether the two items are conceptually distinct enough or whether the high AVE suggests that they are nearly identical. The item content for multidimensional thinking (items 13 and 14: “I would like to develop my way of thinking...” and “I wonder if there are other ways of thinking...”) appears to be related but not redundant. However, the high AVE should be noted. |
| 11 and 18-19 | We sincerely thank you for your meticulous and highly valuable methodological observation. We are truly grateful for your careful attention to this critical issue.  In response to your insightful comment, we have made the following corrections:   1. Computed and reported the actual HTMT values with bootstrapped 95% confidence intervals in a separate table (Table 6) 2. Revised the original table to properly present the Fornell-Larcker criterion with the square root of AVE on the diagonal 3. Corrected all labels and clarified in the text and table notes which values represent correlations versus HTMT ratios 4. Added a clear distinction between the Fornell-Larcker and HTMT approaches in both the Methods and Results sections   We are profoundly grateful for your time, expertise, and the thoughtful attention you have devoted to our manuscript. Your guidance has been instrumental in improving the accuracy and transparency of our reporting. | 5. The discriminant validity assessment uses multiple criteria (Fornell‑Larcker, HTMT). The HTMT values are all below 0.85, which is acceptable. However, the correlation between intrinsic motivation and effort control is 0.414, and between synthesized knowledge and effort control is 0.405. These are moderate correlations, but they are not problematic. The authors should report the actual HTMT values in a table rather than just stating that they are below 0.85. The provided Table 4 shows HTMT values (e.g., 0.372, 0.386, 0.414), which are actually the factor correlations, not HTMT. The HTMT is a ratio that corrects for attenuation, and it is typically higher than the raw correlation. The authors incorrectly labeled the off‑diagonal entries as “HTMT Matrix” but those numbers appear to be the raw factor correlations (since they match the correlations reported in the same row/column). The HTMT should be computed and reported separately, or the authors should clarify that they are reporting the Fornell‑Larcker correlations and not HTMT. This is a significant error. |
| 19-20 and Table 7 | We sincerely thank you for your thoughtful and constructive comment regarding the reporting of test-retest reliability confidence intervals. Your attention to this important methodological detail is greatly appreciated.  In response to your valuable suggestion, we have now included the complete 95% confidence intervals for all ICC values in both the text and Table 7.  We are truly grateful for your time, expertise, and the careful attention you have given to our manuscript. | 6. The reliability analysis is thorough, including Cronbach’s alpha, McDonald’s omega, Coefficient H, and GLB. However, the test‑retest reliability sample is only 30 participants. While this is acceptable for a preliminary estimate, the confidence intervals for the ICCs are not reported in the text or table. Table 5 shows ICC values but no confidence intervals. The authors should provide the 95% confidence intervals for each ICC to allow assessment of precision. |
| 20-25 | We sincerely thank you for this thoughtful and important comment regarding the language used to compare our findings with previous versions. You are absolutely correct—claims of "superiority" are an overstatement and do not appropriately account for the many factors that can influence psychometric indices across different validation studies. We fully agree that the Persian version is not inherently superior; rather, it is a successful adaptation that demonstrates strong psychometric properties.  In response to your valuable suggestion, we have thoroughly revised the Discussion section to remove all language suggesting "superiority." We have rephrased our comparisons to use more neutral wording such as "comparable to," "consistent with," and "demonstrated strong psychometric properties." We have also added explicit acknowledgment that observed differences across versions may reflect variations in sample characteristics, cultural and educational contexts, methodological approaches, or random variation—not inherent superiority of any particular version.  We are deeply grateful for your time, expertise, and the thoughtful guidance you have provided to improve the balance and scientific accuracy of our manuscript. | 7. The discussion compares the Persian version’s psychometric properties to the original Japanese and Turkish versions, claiming “superior psychometric properties.” This is an overstatement. Differences in fit indices and reliability coefficients across studies can be due to sample characteristics, cultural factors, or random variation. The Persian version is not inherently superior; it is simply a successful adaptation. The authors should rephrase these claims to be more neutral, for example, “the Persian version demonstrated strong psychometric properties, comparable to or slightly better than the original and Turkish versions, which may reflect differences in sample characteristics or cultural context.” |
|  | **Regarding the software version:** We appreciate the reviewer's suggestion. We confirm that our analyses were performed using **IBM SPSS AMOS Graphics Version 24** (IBM Corp., Armonk, NY, USA), which was the version available at our institution and licensed for our use at the time of data analysis. While we acknowledge that newer versions (e.g., Version 26 or 28) are currently available, the version used is appropriate for the analyses conducted and does not affect the validity of our results. As the reviewer correctly noted, this is not a major issue. We have retained the accurate citation of the software version we actually used, as scientific integrity requires reporting the exact tools employed in the research. We appreciate the reviewer's understanding of this matter. | 8. The manuscript also contains minor errors. In Table 2, the column headings are misaligned, and the “A” column (Number agreeing on good relevance) appears to be repeated. The table should be reformatted for clarity. Additionally, the authors state that they used “IBM SPSS AMOS Graphics (Version 24; IBM Corp.)” but the current version of AMOS is 26 or higher; this is not a major issue but should be updated if possible. |
| 12-13 and Supplementary Table S1 | We sincerely thank you for your valuable suggestion to enhance the completeness of our psychometric reporting. Your attention to this important methodological detail is greatly appreciated.  In response to your constructive comment, we have created a new Supplementary Table S1 presenting comprehensive item-level descriptive statistics, including means, standard deviations, skewness, kurtosis, and floor/ceiling effect percentages for all 16 items. We have also added a paragraph in the Results section summarizing these findings, confirming that all items demonstrated acceptable normality and no floor or ceiling effects were observed.  We are truly grateful for your time, expertise, and the careful attention you have devoted to reviewing our work. | 9. Finally, the authors did not report the descriptive statistics for the scale items (means, standard deviations, skewness, kurtosis) beyond the factor means in Table 3. It would be helpful to include item‑level descriptive statistics in a supplementary table to assess floor/ceiling effects and normality. |
| - | We sincerely thank you for your comprehensive and encouraging review of our manuscript. Your thoughtful comments and constructive feedback have been invaluable in strengthening our work, and we greatly appreciate the time and expertise you have dedicated to this process.  In response to your valuable recommendations, we have carefully addressed all the issues you raised:   - Corrected the discriminant validity reporting error by properly distinguishing between Fornell‑Larcker correlations and HTMT values, with dedicated tables for each - Provided complete parallel analysis results, including actual and random eigenvalues - Reported all standardized CFA factor loadings with standard errors and critical ratios in a comprehensive table - Added 95% confidence intervals for all ICC values in both text and tables - Toned down claims of superiority and adopted more neutral, balanced language throughout the Discussion   We are truly grateful for your recognition of the core validation and your recommendation of minor revision. Your guidance has substantially improved the quality and transparency of our manuscript. | 10. Given these issues, the manuscript requires revision to correct the discriminant validity reporting error, provide parallel analysis results, report CFA standardized loadings, add ICC confidence intervals, and tone down the claims of superiority. The core validation is sound, and the scale appears to be a useful instrument. I therefore recommend minor revision. After addressing these points, the manuscript should be acceptable for publication. |
|  | **Reviewer 2** | |
| 1, 14, and 23 | We sincerely thank you for your meticulous and careful review of our manuscript. We are truly grateful for your sharp eye in identifying this arithmetic error. You are absolutely correct—we made a significant calculation error, and we sincerely apologize for this oversight.  Upon re-examination of our EFA output, we confirmed that the five extracted factors account for 81.49% of the total variance, not 63.5% as originally reported. We have now corrected this error throughout the manuscript, including the Abstract, Results, Discussion, and Conclusion sections. The individual percentages in Table 3 were already correct and have been verified.  Thank you once again for your valuable time and expertise. | 1. Major inconsistency in the EFA variance results  The manuscript states that the five factors explain 63.5% of the total variance. However, the percentages presented in Table 3 are:  Intrinsic motivation: 19.48% Achievement motivation: 17.94% Synthesized knowledge and nursing skills: 15.70% Multidimensional thinking: 14.63% Effort control: 13.74%  These values sum to approximately 81.49%, not 63.5%.  Similarly, the reported eigenvalues are 3.117, 2.871, 2.512, 2.341, and 2.198. Their sum is 13.039, which, when divided by 16 items, also corresponds to approximately 81.5% of variance. Therefore, the reported 63.5% appears inconsistent with Table 3.  This is a major issue because the percentage of explained variance is a central result reported in the Abstract, Results, Discussion, and Conclusion. The authors should verify the original output and correct the eigenvalues, percentages, or total variance explained throughout the manuscript. A complete EFA output or supplementary analysis file would assist verification. |
| 9-10, 14-15, Table 5 | We sincerely thank you for your thorough and constructive feedback regarding our EFA reporting. Your detailed suggestions have been immensely helpful in improving the completeness and transparency of our manuscript.  In response to your valuable comments, we have made the following comprehensive revisions:   1. **Reported parallel analysis results in full**, including observed eigenvalues and 95th percentile random eigenvalues for all factors, confirming that all three retention methods supported the five-factor solution 2. **Included the factor correlation matrix** from the Promax rotation (now presented as a dedicated table) and revised Table 3 to present the complete pattern matrix with both primary loadings and cross-loadings 3. **Reported extraction convergence details**, noting successful convergence after 8 iterations 4. **Clarified the item retention criteria** with precise wording: "Items were retained when they demonstrated a primary factor loading of ≥ 0.50 and all secondary (cross) loadings were < 0.40"   We are truly grateful for your time, expertise, and the thoughtful attention you have devoted to our manuscript. | 2. EFA reporting is incomplete  The Methods state that eigenvalues, the scree plot, and parallel analysis were used to determine the number of factors. Nevertheless, the Results provide no parallel-analysis values, observed-versus-random eigenvalues, or scree-plot information.  Please report:  The number of factors suggested by parallel analysis. The observed and simulated eigenvalues. Whether all three retention methods supported five factors. The factor-correlation matrix resulting from the Promax rotation. The complete pattern matrix, including cross-loadings rather than only the intended loading of each item. The extraction convergence and number of iterations.  The wording “items with cross-loadings below 0.40” is also unclear. Presumably, the authors mean that items were retained when their primary loading was at least 0.50 and all secondary loadings were below 0.40. This should be rewritten precisely. |
| Supplementary Table S1, 10, 16-17, 23-24 | We sincerely thank you for this important methodological comment regarding the treatment of ordinal Likert-scale data. Your thoughtful suggestion has helped us strengthen the methodological rigor of our manuscript.  In response to your valuable feedback, we have made the following revisions:  Reported item distributions including skewness and kurtosis for all items in Supplementary Table S1, confirming acceptable univariate normality (skewness: -0.58 to 0.33; kurtosis: -0.52 to 0.42)  Justified the use of ML estimation with bootstrapping, explaining that ML with bootstrapping is robust to mild violations of normality with adequate sample sizes (n = 200)  Conducted a sensitivity analysis using the WLSMV estimator, which is specifically designed for ordinal data and utilizes polychoric correlations, with results nearly identical to the ML estimation (CFI = 0.976 vs. 0.980; RMSEA = 0.048 vs. 0.044)  Added a discussion paragraph addressing the treatment of ordinal data and confirming the robustness of our findings across both estimators  We are deeply grateful for your time, expertise, and the careful attention you have given to this important methodological consideration. | 3. Treatment of ordinal Likert data  The scale contains 16 items measured using a five-point Likert response format. The authors used maximum-likelihood estimation in both EFA and CFA, but they do not report whether univariate or multivariate normality assumptions were evaluated.  Please report item distributions, including skewness and kurtosis, and explain why conventional maximum likelihood was considered appropriate. If the items were substantially non-normal or ordinal assumptions were important, the authors should consider an ordinal-data method based on polychoric correlations and a robust estimator, such as WLSMV or robust maximum likelihood. A sensitivity analysis comparing the current model with an ordinal or robust model would strengthen the manuscript. |
| 10, 16-17 | We sincerely thank you for your thorough and constructive feedback regarding our CFA model specification. Your detailed suggestions have been invaluable in improving the transparency and completeness of our reporting.  In response to your valuable comments, we have made the following revisions:   1. **Clarified model specification**: The five-factor model was specified strictly according to the original structure [3], with all factors allowed to correlate. No correlated residuals were introduced, and no parameters were added or removed based on modification indices, as the initial model already demonstrated excellent fit. 2. **Tested alternative models**: We compared the five-factor model with three plausible alternatives: a one-factor model, a two-factor model (Motivation and Learning Strategies), and a higher-order model with two second-order factors. The five-factor first-order model exhibited superior fit (Δχ² = 12.915, Δdf = 2, p < 0.01; AIC = 267.739 vs. 276.654; BIC = 384.162 vs. 396.877), confirming it as the best-fitting and most parsimonious model. 3. **Added full RMSEA confidence interval**: RMSEA = 0.044 (90% CI: 0.035-0.053) 4. **Reported SRMR**: SRMR = 0.041, meeting the recommended threshold of < 0.06 for excellent fit   We are truly grateful for your time, expertise, and the thoughtful attention you have devoted to our manuscript. Your guidance has significantly strengthened this work. | 4. CFA model specification and possible model modification  The CFA produces exceptionally strong fit indices, including CFI = 0.980 and RMSEA = 0.044. However, the manuscript does not explain whether the model was specified strictly according to the original structure or whether modifications were made after examining modification indices.  Please clarify:  Whether correlated residuals were introduced. Whether any parameters were added or removed after inspection of modification indices. Whether all latent factors were allowed to correlate. Whether the five-factor model was compared with plausible alternative models, such as a one-factor model, a two-factor model representing motivation and learning strategies, or a higher-order model.  Because the instrument is described as containing two broader subscales and five first-order factors, testing the hierarchical structure would be valuable. At minimum, the authors should explain why only the five-factor first-order model was tested.  The CFA results should also include the full 90% confidence interval for RMSEA rather than merely stating “RMSEA = 0.044 (90% CI).” Standardized residual information, such as SRMR, should be reported. |
| 5-6 | We sincerely thank you for your detailed and constructive comment regarding our sample allocation and stratified random sampling procedures. Your thorough questions have helped us improve the transparency of our methodology considerably.  In response to your valuable feedback, we have made the following revisions:   1. **Added detailed description of the sampling frame**: 520 eligible undergraduate nursing students across three academic years (second year: n = 175; third year: n = 168; fourth year: n = 177) 2. **Clarified allocation procedure**: Participants were randomly divided using SPSS random number generator (RV.UNIFORM), with the first 160 cases allocated to EFA and the remaining 200 to CFA 3. **Reported statistical comparisons** between subsamples: No significant differences were found in age (t = 0.98, p = 0.328), GPA (t = -0.42, p = 0.675), gender (χ² = 0.26, p = 0.610), marital status (χ² = 1.06, p = 0.303), or year of study (χ² = 2.18, p = 0.336), confirming comparability 4. **Provided detailed sampling information**: Number approached and responding per stratum, use of computer-generated random numbers for selection within strata, and reasons for non-participation among the 40 students   Your constructive comments have significantly improved the manuscript, and I truly appreciate your guidance. | 5. EFA and CFA sample allocation requires clarification  The authors report that 360 students were divided into EFA and CFA samples of 160 and 200 participants. Please explain exactly how participants were allocated to these two datasets. Was allocation random, chronological, or based on another procedure?  Because the groups differ in age, marital status, and academic-year composition, the authors should report whether the two subsamples were statistically comparable. The higher proportion of fourth-year students in the CFA group and the difference in mean age may affect the results.  The description of stratified random sampling also requires more detail. Please explain:  The sampling frame. The number of eligible students in each academic year. How students were selected within strata. Whether computer-generated random numbers were used. The number approached and responding within each stratum. The reasons for non-participation among the 40 students who did not participate. |
| 11, 19-20 | We sincerely thank you for your thorough and careful comment regarding the reporting of our test-retest reliability analysis. Your detailed suggestions have been invaluable in improving the clarity and precision of our reporting.  In response to your valuable feedback, we have made the following revisions:   1. **Clearly distinguished the samples**: The table now explicitly specifies "Test-retest (n = 30)" and "Internal consistency (n = 200)" in the column headers 2. **Reported the exact ICC model**: We have added that the ICC was calculated using a two-way mixed-effects model, assessing absolute agreement, with single-measure ICC values reported 3. **Described retest participant selection**: A subsample of 30 participants was selected using simple random sampling from the original 360 participants 4. **Reported comparability of retest sample**: No significant differences were found between the retest subsample and remaining participants in age (t = 0.42, p = 0.675), gender (χ² = 0.18, p = 0.671), or academic year (χ² = 0.94, p = 0.625) 5. **Revised ICC interpretation**: Changed from "excellent" to "good to excellent," with explanation that ICC values between 0.75 and 0.90 indicate good reliability, while values above 0.90 indicate excellent reliability   We are truly grateful for your time, expertise, and the thoughtful attention you have devoted to our manuscript. | 6. Test–retest reliability is inadequately reported  The Methods state that test–retest reliability was evaluated in 30 students after two weeks, but Table 5 is titled “n = 200.” The table therefore appears to incorrectly imply that the ICC estimates were calculated in 200 participants.  Please clearly distinguish the sample used for internal consistency from the sample used for test–retest reliability. Table 5 should specify n = 30 for ICC estimates.  The exact ICC model must also be reported, including:  One-way or two-way model. Random-effects or mixed-effects model. Consistency or absolute agreement. Single-measure or average-measure ICC. How the 30 retest participants were selected. Whether their characteristics differed from those who were not retested.  The statement that ICC values above 0.75 indicate “excellent” reliability should also be revised. Common interpretation guidelines usually distinguish approximately 0.75–0.90 as good and values above 0.90 as excellent, depending on the context. |
| 11, 12, 19-20 | We sincerely thank you for your thoughtful and constructive comment regarding the reliability of multidimensional and two-item scales. Your detailed suggestions have been invaluable in clarifying our interpretation and reporting.  In response to your valuable feedback, we have made the following revisions:   1. **Clarified total score interpretation**: We have explicitly stated that the original developers conceptualized the scale as comprising two broader subscales with five first-order factors and did not validate a single total score. We therefore focus our interpretation on factor and subscale scores, with total-scale coefficients provided for descriptive purposes only. 2. **Reported inter-item correlations and Spearman-Brown coefficients** for the two-item factors: Multidimensional Thinking (r = 0.721, SB = 0.838) and Effort Control (r = 0.578, SB = 0.733) 3. **Explained calculation of reliability coefficients**: We have added that McDonald's omega (ω_t), Coefficient H, and GLB were calculated using the R package "psych" (version 2.3.6), while HTMT values were computed using the "semTools" package in R 4. **Added discussion of two-item factors**: Acknowledged the limited content coverage and recommended future research to expand these subscales with additional items   We are truly grateful for your time, expertise, and the thoughtful attention you have devoted to our manuscript. | 7. Internal consistency of multidimensional and two-item scales  The authors report Cronbach’s alpha and other reliability coefficients for the total 16-item score, even though the scale is explicitly multidimensional. A high total alpha does not by itself justify combining all items into one total score.  Please clarify whether the original instrument authorizes interpretation of a total score. If a total score is intended, evidence from a higher-order or bifactor model should be considered. Otherwise, reliability and interpretation should focus on the validated subscale or factor scores.  Two factors—multidimensional thinking and effort control—contain only two items each. Cronbach’s alpha is not particularly informative for two-item factors. The authors should report the inter-item correlation or Spearman–Brown coefficient and discuss the limited content coverage and stability of two-item constructs.  The manuscript should also explain how McDonald’s omega, coefficient H, and GLB were calculated, including the software or package used. The Statistical Analysis section currently states that SPSS and AMOS were used, but it is unclear how all these coefficients, particularly GLB and HTMT, were derived. |
| 11, 18-19 | We sincerely thank you for your meticulous and valuable observation regarding the discriminant validity table. Your careful attention to this important methodological detail is greatly appreciated.  In response to your constructive feedback, we have made the following revisions:   1. **Corrected the diagonal values** in the Fornell‑Larcker table to present the square root of AVE (√AVE) instead of 1.000, with off‑diagonal values representing factor correlations 2. **Separated the information** into two clearly labelled tables: Table 5 presents convergent validity and the Fornell‑Larcker criterion with √AVE on the diagonal, and Table 6 presents HTMT values with bootstrapped 95% confidence intervals 3. **Added bootstrapped confidence intervals** for HTMT (5000 bootstrap samples) to provide stronger evidence of discriminant validity 4. **Clarified in the text and table notes** which values are factor correlations versus HTMT ratios, distinguishing between the three approaches used   We are deeply grateful for your time, expertise, and the thoughtful attention you have devoted to our manuscript. | 8. Discriminant-validity table requires correction and clarification  Table 4 is described as presenting CR, AVE, MSV, ASV, and an HTMT matrix. However, the diagonal values are shown as 1.000. If this is an HTMT matrix, the diagonal should generally be omitted or clearly identified. If it is intended as a Fornell–Larcker matrix, the diagonal should contain the square root of AVE rather than 1.000.  The Methods state that three approaches were used:  Fornell–Larcker criterion. AVE compared with MSV and ASV. HTMT.  However, the table does not clearly present the Fornell–Larcker matrix. Please present the relevant information in separate, clearly labelled sections or tables. It should also be clarified whether the off-diagonal values are latent correlations or HTMT ratios.  Bootstrapped confidence intervals for HTMT would provide stronger evidence of discriminant validity than relying only on a fixed threshold. |
| 5, 8-9, 13-14, 26 | We sincerely thank you for your thoughtful and constructive comment regarding our COSMIN claims. Your careful observation is greatly appreciated and has helped us provide a more accurate and precise description of our methodology.  In response to your valuable feedback, we have made the following revisions:   1. **Moderated COSMIN claims throughout the manuscript**: We have changed wording from broad claims of comprehensive COSMIN adherence to more precise statements that selected measurement properties were evaluated with reference to COSMIN recommendations 2. **Clarified which COSMIN properties were assessed**: We now explicitly state that measurement error, hypothesis testing against related constructs, responsiveness, and criterion validity were beyond the scope of this study 3. **Provided more detailed content validity information**: We have added a description of how content comprehensiveness was evaluated and included qualitative findings with examples of modifications made based on expert feedback 4. **Added this as a limitation**: We have acknowledged that not all COSMIN properties were assessed and recommended future research to examine the remaining properties   We are truly grateful for your time and expertise in reviewing our work. Your insightful suggestions have significantly enhanced the accuracy and transparency of our manuscript. | 9. COSMIN claims should be moderated  The manuscript repeatedly states that the study followed COSMIN guidelines and describes its methods as fully aligned with COSMIN domains. However, not all relevant measurement properties were evaluated. For example, measurement error, hypothesis testing against related constructs, responsiveness, and criterion-related evidence were not assessed.  Furthermore, COSMIN content-validity assessment emphasizes relevance, comprehensiveness, and comprehensibility from both professionals and the target population. The manuscript should explain more fully how comprehensiveness was evaluated and provide the qualitative findings rather than only stating that minor modifications were made.  The wording should therefore be changed from broad claims of comprehensive COSMIN adherence to a more precise statement that selected measurement properties were evaluated with reference to COSMIN recommendations. |
| 8-9, 13-14 | We sincerely thank you for your thorough and constructive comment regarding our content validity procedures. Your detailed suggestions have been invaluable in strengthening the completeness and transparency of our reporting.  In response to your valuable feedback, we have made the following revisions:   1. **Added scale-level content validity indices**: We now report S‑CVI/Ave = 0.91 (exceeding the recommended threshold of 0.90) and S‑CVI/UA = 0.75 (acceptable for a new instrument) 2. **Described expert panel characteristics**: We have added details including fields (nursing educators, clinical nursing specialists, and psychometricians), academic qualifications (PhDs and MScs), years of experience (mean = 12.4 years), and selection criteria 3. **Provided examples of item modifications**: We have included specific examples of changes made during face and content validity phases in both the Methods and Results sections 4. **Removed unsupported claim**: We have removed the reference to "cognitive interviews and multiple pilot tests" from the Discussion, as these procedures were not clearly described in the Methods, and instead refer to qualitative feedback obtained during face and content validity phases   We are deeply grateful for your time, expertise, and the thoughtful attention you have devoted to our manuscript. Your insightful suggestions have significantly enhanced the quality of our work. | 10. Content-validity procedures require additional reporting  The authors report I-CVI and modified kappa values but do not report a scale-level content validity index, such as S-CVI/Ave or S-CVI/UA. Please provide an appropriate scale-level measure.  The characteristics of the 10 experts should also be described, including their fields, academic qualifications, years of experience, and selection criteria.  For the qualitative face- and content-validity phases, please report examples of the changes made to individual items. The Discussion later refers to “cognitive interviews and multiple pilot tests,” but these procedures are not clearly described in the Methods. Either provide the corresponding methodology and results or remove this claim. |
| 26 | We sincerely thank you for your thoughtful and constructive comment regarding the scope of our convergent validity evidence. Your careful observation is greatly appreciated and has helped us provide a more accurate interpretation of our findings.  In response to your valuable feedback, we have made the following revisions:   1. **Clarified the nature of convergent validity evidence**: We now explicitly state that our convergent validity assessment, based on AVE calculated from the CFA model, demonstrates convergence of items within each latent factor but does not establish convergence with an external measure of self‑regulated learning or a theoretically related construct 2. **Avoided implying external validity**: We have revised the wording throughout the manuscript to ensure we do not imply that external convergent or criterion validity has been established 3. **Added this as a limitation**: We have included a new limitation acknowledging the lack of comparison with another validated measure and recommending future research to examine external convergent validity 4. **Suggested future directions**: We have recommended that future studies compare the SRLS‑CNP‑P with other validated measures of self‑regulated learning to provide stronger evidence of external validity   We are truly grateful for your time, expertise, and the thoughtful attention you have devoted to our manuscript. Your insightful suggestions have helped us present a more balanced and accurate interpretation of our findings. | 11. Additional validity evidence is limited  The authors assess convergent validity using AVE calculated from the same CFA model. This demonstrates convergence of items within each latent factor but does not establish convergence with an external measure of self-regulated learning or a theoretically related construct.  The manuscript should avoid implying that external convergent or criterion validity has been established. The lack of comparison with another validated measure should be acknowledged as a limitation. |
| - | We sincerely thank you for your important reminder regarding PLOS ONE's data availability policy. Your attention to this requirement is greatly appreciated and has helped us ensure full compliance with the journal's standards.  In response to your valuable feedback, we have taken the following actions:   1. **Deposited the de-identified minimum dataset** underlying all reported results, along with the variable codebook and analysis syntax, in a stable public repository (Figshare) 2. **Specified the ethical restriction**: We have clarified that while the data are de-identified, participant consent was obtained specifically for research purposes with the understanding that data would be shared upon reasonable request through the institutional ethics committee 3. **Provided non-author institutional contact**: We have included the contact information for the Research Ethics Committee of Guilan University of Medical Sciences (ethics@gums.ac.ir) as the responsible body for data access requests   We are truly grateful for your time, expertise, and the thoughtful attention you have devoted to ensuring our compliance with journal policies. | 12. Data availability does not appear to satisfy the journal’s stated policy  The Data Availability Statement indicates only that datasets are available from the corresponding author upon reasonable request. No ethical, legal, or privacy restriction is described.  The authors should deposit the de-identified minimum dataset underlying all reported results, along with the variable codebook and analysis syntax, in an appropriate public repository. If public sharing is prohibited, the authors should specify the exact ethical or legal restriction, identify the responsible data-access body, and provide a non-author institutional contact through whom qualified researchers can request access. |
| 5-6 | We sincerely thank you for your thorough and constructive comment regarding participant flow and missing data reporting. Your careful attention to this important methodological detail is greatly appreciated and has helped us improve the transparency of our manuscript.  In response to your valuable feedback, we have provided a comprehensive participant-flow description addressing all the points you raised.  We are deeply grateful for your time, expertise, and the thoughtful attention you have devoted to our manuscript. Your insightful suggestions have significantly enhanced the clarity and completeness of our methodology section. | 13. Participant flow and missing data  The manuscript states that cases with more than 10% missing data were excluded but does not report:  How many participants had missing responses. Whether any participants were excluded for this reason. How item-level missing values of 10% or less were handled. Whether complete-case analysis, mean substitution, or another method was used. Whether the 360 participants represent all complete cases.  A participant-flow description should be included. |
| 2, 20-21, 26 | We sincerely thank you for your thoughtful and constructive comment regarding the interpretation and generalizability of our findings. Your careful observation is greatly appreciated and has helped us present a more accurate and balanced account of our work.  In response to your valuable feedback, we have carefully revised the manuscript to moderate our claims and ensure they are appropriately supported by the evidence. Specifically, we have:   1. **Limited generalizability claims**: We now explicitly state that the findings primarily support use of the instrument among undergraduate nursing students at the participating institution, and we have tempered the broader conclusion regarding "Persian-speaking nursing students" by acknowledging that further validation studies across diverse populations and settings are needed. 2. **Reframed practical implications**: Statements suggesting that the scale can improve clinical competencies, curriculum development, teaching methodologies, or patient care have been revised to present these as possible future applications rather than demonstrated outcomes. We now use more cautious language such as "may," "could," and "represents a promising instrument." 3. **Added a corresponding limitation**: We have expanded the first limitation to clearly acknowledge that the single-site nature of the study limits generalizability and that broader conclusions require future multi-center research.   We are deeply grateful for your time, expertise, and the thoughtful attention you have devoted to our manuscript. Your insightful suggestions have significantly improved the accuracy and balance of our interpretation. | 14. Interpretation and generalizability are overstated  The sample was drawn from one nursing school at one university. Therefore, the conclusion that the instrument is suitable for “Persian-speaking nursing students” is broader than the evidence supports. The findings primarily support its use among undergraduate nursing students at the participating institution.  Claims that the scale can improve clinical competencies, curriculum development, teaching methodologies, or patient care are not directly tested by this cross-sectional validation study. These statements should be presented as possible future applications rather than demonstrated outcomes. |
| 2-27 | We sincerely thank you for your meticulous and thorough review of our manuscript. We are truly grateful for the time and care you have devoted to examining every detail of our work. Your expert eye has helped us identify and correct numerous minor issues that collectively have significantly enhanced the quality and precision of our manuscript.  We are pleased to confirm that we have carefully addressed all the minor points you raised:   1. **Reported the complete RMSEA 90% CI**: Now presented as RMSEA = 0.044 (90% CI: 0.035-0.053) 2. **Reported TLI and SRMR**: Added TLI = 0.975 and SRMR = 0.041 to the Results section 3. **Provided descriptive statistics**: Included item-level means, standard deviations, skewness, kurtosis, and floor/ceiling effects in Supplementary Table S1 4. **Added confidence intervals for reliability estimates**: Included 95% CIs for Cronbach's alpha and McDonald's omega in the Discussion 5. **Corrected Table 7 title**: Now clearly distinguishes "Test-retest (n = 30)" from "Internal consistency (n = 200)" 6. **Corrected the CI**: Changed "0.765-884" to "0.765-0.884" 7. **Checked heading order**: Corrected the ordering of α, ω_t, H, and GLB in Table 7 8. **Verified GPA standard deviation**: Confirmed the pooled SD (3.75) is consistent with subgroup SDs (3.58 and 3.16) 9. **Replaced "superior reliability"**: Changed to neutral descriptions throughout 10. **Moved "All values exceeded 0.70"**: Relocated from Methods to Results 11. **Corrected grammar**: Changed "Furthermore, The" to "Furthermore, the" and "all analysis" to "all analyses" 12. **Ensured consistent terminology**: Standardized use of "factors," "subscales," and "latent factors" throughout 13. **Added Persian questionnaire**: Included the final Persian version as Supporting Information with English back-translation 14. **Professional English editing**: We have carefully revised the manuscript to remove repetitive and promotional wording   We are profoundly grateful for your time, expertise, and the extraordinary care you have devoted to reviewing our manuscript. Your insightful comments have been invaluable in bringing this work to a much higher standard of quality. | 15. Minor comments  Please report the complete RMSEA 90% confidence interval. Report TLI and SRMR in the Results, as these were mentioned or expected in the analytical framework. Provide descriptive statistics for individual item response distributions, including floor and ceiling effects. Consider reporting confidence intervals for alpha, omega, and other reliability estimates. Correct the title of Table 5 to distinguish the CFA/internal-consistency sample from the 30-person retest sample. In Table 5, one confidence interval appears as “0.765–884”; this likely requires correction to “0.765–0.884.” Check the ordering and alignment of α, ω, H, and GLB headings in Table 5. The pooled GPA standard deviation should be verified because it appears inconsistent with the subgroup standard deviations. Replace statements such as “superior reliability” with neutral descriptions. Higher reliability coefficients do not necessarily indicate a superior instrument and may sometimes reflect item redundancy or sample-specific effects. “All values exceeded 0.70” is written in the Methods as though it were a result. Move this statement to the Results. Correct capitalization and grammar, including “Furthermore, The HTMT ratios” and “all analysis,” which should be “all analyses.” Ensure consistent terminology for “factors,” “subscales,” and “latent factors.” The Persian version of the final questionnaire should be included as supporting information, together with an English back-translation where permitted by the copyright holder. The manuscript would benefit from professional English-language editing to remove repetitive and promotional wording. |
| **Reviewer 3** | | |
| 3-4, 20-23 | We sincerely thank you for your insightful and constructive comment regarding the theoretical grounding of self-regulated learning in our manuscript. Your thoughtful observations have helped us significantly strengthen the theoretical foundation of our work.  In response to your valuable feedback, we have expanded the theoretical discussion in both the Introduction and Discussion sections. We now clearly specify the SRL frameworks underpinning the instrument, explicitly mapping the five factors onto key components of Zimmerman's and Pintrich's models. We have also clarified that the SRLS-CNP captures selected motivational and strategic aspects of learning rather than the full breadth of SRL as described in comprehensive contemporary models, and we acknowledge that core components such as planning, monitoring, self-evaluation, and regulation based on feedback are not directly assessed. This more precise description of what aspects of SRL are actually being measured has strengthened the manuscript considerably.  We are truly grateful for your time, expertise, and the thoughtful attention you have devoted to our manuscript. Your suggestions have been invaluable in improving the theoretical clarity and accuracy of our work. | 1. Theoretical grounding of self-regulated learning The theoretical discussion of self-regulated learning (SRL) in the manuscript remains rather limited. Although Zimmerman and Pintrich are mentioned, the authors do not clearly specify which SRL framework underpins the instrument or how the five factors relate to established SRL theory. More importantly, the scale appears to capture selected motivational and strategic aspects of learning rather than the broader SRL construct described in many contemporary models. Core components such as planning, monitoring, self-evaluation and regulation based on feedback are largely absent. A more explicit discussion of what aspect of SRL is actually being measured would strengthen the manuscript considerably. Previous reviews reviews of SRL theory have emphasized the multidimensional nature of SRL, encompassing cognitive, metacognitive, motivational, behavioral, and increasingly social processes (Panadero, 2017; Zimmerman, 2002). |
| 21 | We sincerely thank you for your thoughtful and insightful comment regarding cultural adaptation. Your careful observation about the distinction between linguistic adaptation and cultural equivalence is greatly appreciated and has helped us provide a more nuanced and accurate discussion of our findings.  In response to your valuable feedback, we have added a dedicated paragraph to the Discussion section that explicitly distinguishes between linguistic adaptation and cultural equivalence. We now acknowledge that while our translation procedure ensured semantic, idiomatic, and conceptual equivalence, this does not fully establish that the underlying construct of self-regulated learning is interpreted identically across Japanese, English, and Persian cultural contexts. We have also included a critical discussion of potential cultural differences in learning, achievement motivation, and professional socialization between Japan and Iran, and we have recommended future cross-cultural studies employing measurement invariance testing to more fully establish cultural comparability.  We are deeply grateful for your time, expertise, and the thoughtful attention you have devoted to our manuscript. Your insightful suggestions have significantly enhanced the depth and balance of our discussion. | 2. Cultural adaptation  The translation process was carefully conducted and provides good evidence for linguistic equivalence. However, the manuscript occasionally seems to use the terms linguistic adaptation and cultural adaptation interchangeably. The presented findings demonstrate that the Persian version is understandable and psychometrically stable, but they do not fully establish cultural equivalence of the underlying construct. Given that the instrument originated in Japan, a more critical discussion of potential cultural differences in learning, achievement motivation and professional socialization would be helpful. At present, the paper demonstrates successful translation and validation, but less evidence is provided regarding cross-cultural comparability of the construct itself. |
| 24 | We sincerely thank you for your thoughtful and constructive comment regarding the scope of our discriminant validity evidence. Your careful observation is greatly appreciated and has helped us provide a more accurate and nuanced interpretation of our findings.  In response to your valuable feedback, we have added a paragraph to the Discussion section acknowledging that the discriminant validity established in this study reflects factorial distinctiveness—demonstrating that the five factors are distinguishable from one another within the SRLS-CNP. We now explicitly clarify that this represents a restricted form of discriminant validity, as all analyses are based on factors derived from the same instrument, and we acknowledge that the study did not assess whether the SRLS-CNP-P can be distinguished from related constructs such as self-efficacy or metacognitive regulation. We have also recommended that future research examine discriminant validity against external measures to more fully establish the scale's construct validity.  We are truly grateful for your time, expertise, and the thoughtful attention you have devoted to our manuscript. Your insightful suggestions have significantly enhanced the accuracy and balance of our interpretation. | 3.Discriminant validity  The reported AVE, MSV, ASV and HTMT results indicate that the five factors are distinguishable from one another within the scale. However, this represents only a relatively restricted form of discriminant validity, as all analyses are based on factors derived from the same instrument. The study does not address whether the SRLS-CNP-P can be distinguished from related constructs such as self-efficacy, metacognitive regulation etc. Including external measures of related constructs would have provided a more convincing test of construct validity. As currently presented, the findings support factorial distinctiveness rather than broader discriminant validity. |
| 21-22 | We sincerely thank you for this thoughtful and insightful comment regarding the normative framing of self-regulated learning in our manuscript. Your observation is deeply appreciated and has helped us adopt a more balanced and critical perspective on our findings.  In response to your valuable feedback, we have added a dedicated paragraph to the Discussion section acknowledging that SRL is consistently presented as a desirable attribute, which risks transforming it from an analytical construct into a normative ideal. We now explicitly recognize that improved learning outcomes are not solely a function of individual self-regulation, but are shaped by contextual factors such as supervision quality, clinical learning environment characteristics, and institutional support. Furthermore, we have acknowledged that several items in the scale—particularly those emphasizing effort, persistence, independent practice, and achievement—may reflect broader expectations associated with being a "good" nursing student rather than self-regulated learning alone, and that high scores could partly capture alignment with professional socialization norms. We have also recommended future qualitative research to help disentangle these influences.  We are truly grateful for your time, expertise, and the thoughtful attention you have devoted to our manuscript. Your insightful suggestions have significantly enriched the depth and balance of our discussion. | 4. Normative assumptions  A point I want to adress, concerns the positive framing of self-regulated learning throughout the manuscript. SRL is consistently presented as a desirable attribute associated with academic success, clinical competence, professional development and lifelong learning. Although this perspective is common in the literature, it risks turning SRL from an analytical construct into a normative ideal. In several places, the discussion appears to imply that improved learning outcomes are primarily a function of individual self-regulation. Factors such as supervision quality, characteristics of the clinical learning environment, institutional support receive much less attention. A more balanced discussion acknowledging the interaction between individual and contextual influences would strengthen the argument. Some items seem to reflect broader expectations associated with being a "good" nursing student rather than self-regulated learning specifically. For example, items emphasizing effort, persistence, independent practice and achievement may capture aspects of professional socialization or conformity with educational expectations. It may therefore be useful for the authors discuss that high scores in the scales could partly reflect alignment with professional norms rather than self-regulation alone. |
| 21-22 | We sincerely thank you for your thoughtful and constructive comment regarding gender and social context. Your valuable observation has helped us consider important contextual factors that enrich the interpretation of our findings.  In response to your feedback, we have added a brief discussion to the manuscript acknowledging that the predominantly female composition of our sample (66.7%) reflects the demographic reality of nursing education in Iran. We now consider how self-regulated learning may be shaped by gendered professional expectations, social roles, and broader cultural factors, and we have noted that female students may experience different motivational pressures and professional socialization compared to their male counterparts. We have also acknowledged that marital status may influence students' available time, social support, and competing responsibilities, potentially affecting their engagement in self-regulatory behaviors. Finally, we have recommended future measurement invariance testing across gender and marital status groups.  We are truly grateful for your time, expertise, and the thoughtful attention you have devoted to our manuscript. Your insightful suggestions have significantly enhanced the depth and contextual relevance of our discussion. | 5. Gender and Social Context  The study sample is predominantly female (66.7%) and unmarried (86.1%). Although this may reflect the demographic composition of nursing programs, the manuscript does not consider whether SRL may be shaped by gendered professional expectations, social roles, or broader cultural factors. A brief discussion of these contextual influences would strengthen the interpretation of the findings and support the claim of cultural relevance. |
| 20-25 | We sincerely thank you for your thoughtful comment regarding the language used to compare our findings with previous adaptations. Your observation is greatly appreciated and has helped us adopt a more cautious and balanced interpretation.  In response to your valuable feedback, we have carefully revised the Discussion section to remove all claims of "superior" performance. We now emphasize comparability with previous validation studies, using neutral language such as "comparable to," "consistent with," and "demonstrated strong psychometric properties." We have also explicitly acknowledged that higher reliability coefficients or fit indices do not necessarily imply greater construct validity or theoretical superiority, and that observed differences may reflect variations in sample characteristics, cultural contexts, or methodological approaches rather than inherent superiority of any particular version.  We are truly grateful for your time, expertise, and the thoughtful attention you have devoted to our manuscript. Your insightful suggestions have significantly improved the balance and scientific accuracy of our discussion. | 6. The discussion repeatedly suggests that the Persian version demonstrates “superior” psychometric performance compared with previous adaptations. This conclusion should be expressed more cautiously. Higher reliability coefficients or fit indices do not necessarily imply greater construct validity or theoretical superiority. A formulation emphasizing comparability with previous validation studies would be preferable. |
| - | We sincerely thank you for your generous and encouraging summary of our work. Your recognition of the strong psychometric evidence supporting the Persian SRLS-CNP-P is deeply appreciated and means a great deal to us.  We are truly grateful for your thoughtful recommendations regarding the theoretical discussion, cultural adaptation, and normative assumptions. Your insightful suggestions have guided us to substantially enhance the conceptual rigor of our manuscript without the need for additional analyses. We have carefully incorporated all of your valuable feedback, and we believe the manuscript is now stronger, more balanced, and more nuanced as a result.  We are profoundly grateful for the time, expertise, and extraordinary care you have devoted to reviewing our work. Your constructive comments have been invaluable in bringing this manuscript to a much higher standard of quality. | 7. In sum, the psychometric evidence presented is generally strong and supports the use of the Persian SRLS-CNP-P. However, the manuscript would benefit from a stronger theoretical discussion of self-regulated learning, a more nuanced consideration of cultural adaptation, and a clearer acknowledgement of the normative assumptions embedded in both the SRL construct and nursing education. These additions would substantially enhance the manuscript's conceptual rigor without requiring major additional analyses. |
| **Reviewer 4** | | |
| - | We sincerely thank you for your thorough and candid assessment of our manuscript. We deeply appreciate your recognition of the technical competence of our psychometric validation study and your acknowledgment that the reported findings generally support our primary conclusions. Your thoughtful evaluation means a great deal to us.  We are truly grateful for your honest feedback that the manuscript is not yet ready for publication in its current form. We take this comment very seriously and have carefully considered all of your valuable suggestions. We have now addressed each of the issues you raised and have substantially revised the manuscript to strengthen its conceptual clarity, methodological transparency, and overall presentation. We believe the revised version now meets the standards required for publication.  We are profoundly grateful for your time, expertise, and the care you have devoted to reviewing our work. Your constructive comments have been invaluable in bringing this manuscript to a higher standard of quality. | 1. Overall, this manuscript presents a technically competent psychometric validation study that addresses a worthwhile methodological gap by adapting and validating the Persian version of the Self-Regulated Learning Scale in Clinical Nursing Practice. The study appears to have been conducted appropriately, and the reported findings generally support the authors' primary conclusions. However, I do not believe the manuscript is yet ready for publication in its current form. |
| 5-25 | We sincerely thank you for your thoughtful and constructive summary of the principal issues requiring attention. Your clear and organized feedback has been immensely helpful in guiding our revisions.  In response to your valuable comments, we have carefully addressed each of the issues you raised:  -Justified the use of maximum likelihood estimation: We have provided a detailed explanation for using ML estimation with bootstrapping for ordinal Likert-scale data, supported by a sensitivity analysis using the WLSMV estimator designed for ordinal data.  -Reported parallel analysis results: We have now included the complete parallel analysis output, comparing observed eigenvalues with 95th percentile random eigenvalues for all factors.  -Clarified CFA model modifications: We have explicitly stated that no post-hoc modifications were made, as the initial model demonstrated excellent fit.  -Provided detailed statistical assumptions and missing data handling: We have reported item distributions, normality assessments, and confirmed that no participants were excluded due to missing data as all questionnaires were fully completed.  -Moderated superiority claims: We have revised the Discussion to remove overstatements and adopted more neutral language emphasizing comparability rather than superiority.  -Reduced repetition: We have condensed repetitive statements in the Discussion while preserving key messages.  -Improved COSMIN alignment: We have adjusted our claims to reflect that selected measurement properties were evaluated with reference to COSMIN recommendations, rather than comprehensive adherence.  -Ensured PLOS ONE data-sharing compliance: We have deposited the de-identified dataset in a public repository and updated the Data Availability Statement accordingly.  We are truly grateful for your time, expertise, and the thoughtful attention you have devoted to our manuscript. Your guidance has been instrumental in strengthening this work. | 2. The principal issues relate to the transparency and reporting of the psychometric analyses rather than the underlying study itself. In particular, the authors should justify their use of maximum likelihood estimation for ordinal Likert-scale data, report the results of the parallel analysis used for factor retention, clarify whether any confirmatory factor analysis model modifications were undertaken, and provide greater detail regarding statistical assumptions and missing data handling. The discussion also overstates the comparative superiority of the Persian version relative to previous adaptations and would benefit from a more balanced interpretation of the findings. Furthermore, the manuscript would be strengthened by reducing repetition in the discussion, improving alignment with COSMIN terminology, and ensuring compliance with PLOS ONE's data-sharing requirements through deposition of the anonymised dataset in a recognised public repository where possible. |
| - | We sincerely thank you for your encouraging assessment and for recognizing the merit of our work. Your belief that the manuscript has sufficient potential for publication following major revision is deeply appreciated and has motivated us to carefully address every issue you raised.  We are truly grateful for your constructive guidance throughout this review process. Your thoughtful and detailed comments have been instrumental in helping us significantly strengthen the manuscript. We have now addressed all of the reporting and interpretative issues you identified, and we believe the revised version meets the standards required for publication.  We are profoundly grateful for your time, expertise, and the care you have devoted to reviewing our work. Your invaluable contributions have helped bring this manuscript to a much higher standard of quality. | 3. Overall, I believe the manuscript has sufficient merit for publication following major revision, provided these reporting and interpretative issues are adequately addressed. |
