# Supplementary material for: Psychometric properties of the Persian version of the self-regulated learning scale in clinical nursing practice for nursing students: A methodological study
Source: PLoS One. 2026 Sep 25;21(9):e0358790. doi: 10.1371/journal.pone.0358790 (PMC13614530; doi:10.1371/journal.pone.0358790)
Supplement: Supplementary file 6 [file pone.0358790.s008.docx]

***PLOS ONE***

**Academic Editor**

**Dear Professor De-Chih Lee,**

Thank you for considering the revised version of our manuscript entitled "**Psychometric properties of the Persian version of the self-regulated learning scale in clinical nursing practice for nursing students: A methodological study**." We appreciate the editor and reviewers for highlighting essential modifications required for the manuscript. Their insightful comments have been incredibly valuable in restructuring our work. We have carefully considered these suggestions. We provide a detailed, point-by-point explanation of the changes made in response to the reviewers' feedback on the following pages, with all modifications highlighted in yellow in the manuscript. We sincerely hope these revisions meet the criteria for publication in *PLOS ONE*.

We are looking forward to hearing from you.

| **Page(s)** | **Author response to comments** | **Comments** |
| --- | --- | --- |
|  | **Reviewer 2** | |
| 26 | We sincerely thank the reviewer for this valuable and insightful comment regarding the S-CVI/UA value.  We fully agree with the reviewer that the S-CVI/UA of 0.75 falls below the conventional threshold of ≥0.80. In response to this important observation, we have now explicitly acknowledged this issue as a limitation in the Discussion section of the revised manuscript.  We believe this addition addresses the reviewer's concern transparently and appropriately.  Once again, we are deeply grateful for the reviewer's meticulous and constructive feedback, which has significantly improved the quality and clarity of our manuscript. | 1. Scale-level content validity (S-CVI/UA): You report S-CVI/UA = 0.75, which falls below the conventional threshold of ≥0.80. Please either cite a source justifying this value for a new instrument or briefly acknowledge it as a limitation in the text. |
| 12 and Table 1 | We sincerely thank the reviewer for their meticulous and valuable attention to the numerical accuracy of our data. This kind of careful review is greatly appreciated and has helped us improve the precision of our manuscript.  The reviewer is absolutely correct. Upon re-examining our calculations, we identified a typographical error in the reported total standard deviation for GPA. The value of 3.75 was mistakenly entered due to a data entry oversight. The correct pooled standard deviation, calculated using the comprehensive formula that accounts for both within-group variances and the difference between subgroup means, is **3.35**.  We have now corrected this value in Table 1 and the corresponding text in the Results section. The detailed calculation is as follows:  ${SD}_{pooled}=\sqrt{\frac{(n_{1}-1){SD}_{1}^{2}+(n_{2}-1){SD}_{2}^{2}+\frac{n_{1}n_{2}}{n_{1}+n_{2}}{(\overline{X_{1}}-\overline{X_{2}})}^{2}}{n_{1}+n_{2}-1}}=3.3526\approx3.35$  We are truly grateful to the reviewer for their sharp observation and constructive feedback, which has undoubtedly enhanced the scientific rigor and credibility of our work. | 2. Pooled GPA standard deviation: In Table 1, the EFA and CFA subgroups report SDs of 3.58 and 3.16, yet the total sample SD is reported as 3.75. Given the similar means, the pooled SD should be approximately 3.35. Please verify your calculation and correct this value, or provide the exact method used to derive 3.75. |
| 2 | We sincerely thank the reviewer for their careful attention to this important technical detail. Such meticulous review is invaluable in ensuring the final manuscript is professionally presented and free from any editing artifacts.  The reviewer is entirely correct. During the revision and merging process, a formatting artifact unfortunately remained in the Abstract, resulting in the incorrect value "81.4963.5%." This has now been corrected to the proper value of **"81.49%"** in the final clean version of the manuscript. We have thoroughly reviewed the entire text to ensure that no other merge artifacts remain.  We are truly grateful to the reviewer for pointing out this oversight, which has helped us enhance the overall clarity and professionalism of our manuscript. | 3. Tracked-changes artifacts: Please ensure the final clean version removes all merge artifacts (e.g., "81.4963.5%" in the Abstract). |
| - | We sincerely thank the reviewer for this thoughtful and constructive summary. We truly appreciate the time and expertise devoted to reviewing our work.  We have carefully addressed each of the points raised and have made all the necessary corrections and clarifications throughout the revised manuscript.  We are confident that these revisions have substantially improved the clarity, transparency, and scientific accuracy of our manuscript.  Once again, we are deeply grateful to the reviewer for their meticulous and invaluable feedback, which has significantly strengthened our work. We sincerely appreciate their time and effort. | 4. These require no re-analysis, but they do need correction or brief clarification before the manuscript can be accepted. |
|  | **Reviewer 5** | |
| 26 | We sincerely thank the reviewer for this valuable and insightful comment regarding the S-CVI/UA value.  We fully agree with the reviewer that the S-CVI/UA of 0.75 falls below the conventional threshold of ≥0.80. In response to this important observation, we have now explicitly acknowledged this issue as a limitation in the Discussion section of the revised manuscript.  We believe this addition addresses the reviewer's concern transparently and appropriately.  Once again, we are deeply grateful for the reviewer's meticulous and constructive feedback, which has significantly improved the quality and clarity of our manuscript. | 1. First, the content-validity reporting is still internally inconsistent. In the Methods, the manuscript states that S-CVI/UA should meet an acceptable threshold of ≥0.80, but the Results report S-CVI/UA = 0.75 and still describe the instrument as having strong overall content validity. |
| 12 and Table 1 | We sincerely thank the reviewer for their meticulous and valuable attention to the numerical accuracy of our data. This kind of careful review is greatly appreciated and has helped us improve the precision of our manuscript.  The reviewer is absolutely correct. Upon re-examining our calculations, we identified a typographical error in the reported total standard deviation for GPA. The value of 3.75 was mistakenly entered due to a data entry oversight. The correct pooled standard deviation, calculated using the comprehensive formula that accounts for both within-group variances and the difference between subgroup means, is **3.35**.  We have now corrected this value in Table 1 and the corresponding text in the Results section. The detailed calculation is as follows:  ${SD}_{pooled}=\sqrt{\frac{(n_{1}-1){SD}_{1}^{2}+(n_{2}-1){SD}_{2}^{2}+\frac{n_{1}n_{2}}{n_{1}+n_{2}}{(\overline{X_{1}}-\overline{X_{2}})}^{2}}{n_{1}+n_{2}-1}}=3.3526\approx3.35$  We are truly grateful to the reviewer for their sharp observation and constructive feedback, which has undoubtedly enhanced the scientific rigor and credibility of our work. | 2. Second, the demographic table still appears to contain a numerical inconsistency. The EFA and CFA subgroups report GPA SDs of 3.58 and 3.16, but the total sample SD is reported as 3.75. Given the subgroup means and SDs, the pooled total SD should not normally exceed both subgroup SDs by this amount unless there is a specific calculation issue or data-distribution explanation. |
| Table 5 | We sincerely thank the reviewer for their sharp and careful observation regarding the numerical value in Table 5. We are genuinely grateful for this meticulous attention to detail, which has helped us improve the accuracy of our validity table.  The reviewer is absolutely correct. Upon rechecking our data, we identified a typographical error in the diagonal value for Achievement Motivation. The value was mistakenly entered as "1.847" instead of the correct value, which is **"0.847"** (the square root of AVE = 0.718). This error has now been corrected in Table 5 of the revised manuscript. We have also double‑checked all other diagonal values in the table to ensure their accuracy.  We truly appreciate the reviewer's valuable feedback, which has significantly enhanced the precision and scientific integrity of our work. We are deeply grateful for their time and expertise. | 3. Third, Table 5 contains a clear typographical or numerical error. The diagonal value for Achievement Motivation is reported as 1.847, although the square root of AVE for AVE = 0.718 should be approximately 0.847. This should be corrected, because it appears in a key validity table. |
| Table 7 | We sincerely thank the reviewer for this thoughtful and constructive observation. Their careful attention to the accuracy of our reporting is greatly appreciated.  The reviewer is absolutely correct. The original title of Table 7 ("... (n = 200)") was indeed misleading, as it did not clearly distinguish between the sample sizes used for internal consistency (n = 200) and test-retest reliability (*n* = 30). We have now revised the table title to **"Reliability estimates of the SRLS-CNP-P"** and have clearly labeled the two sections as "Internal Consistency (n = 200)" and "Test-Retest (*n* = 30)."  Additionally, we have added a superscript footnote and a comprehensive note beneath the table to explicitly clarify that ICC estimates were derived from the subsample of 30 participants.  We are truly grateful to the reviewer for this valuable feedback, which has helped us ensure greater transparency and precision in our reporting. | 4. Fourth, the reliability table title still states n = 200, even though the table includes both internal-consistency estimates based on n = 200 and test–retest ICC estimates based on n = 30. The table itself labels the two sections, but the title should be revised to avoid the impression that ICCs were calculated from 200 participants. |
