# Supplementary material for: Psychometric properties of the Persian version of the self-regulated learning scale in clinical nursing practice for nursing students: A methodological study
Source: PLoS One. 2026 Sep 25;21(9):e0358790. doi: 10.1371/journal.pone.0358790 (PMC13614530; doi:10.1371/journal.pone.0358790)
Supplement: Peer review history

# Peer review: Decision Letter 0

**DOI:** 10.1371/journal.pone.0358790.r001

**Authors:** De-Chih Lee

27 Jul 2026

Dear Dr. Nemati-Vakilabad,

Thank you for submitting your manuscript to PLOS ONE. After careful consideration, we feel that it has merit but does not fully meet PLOS ONE’s publication criteria as it currently stands. Therefore, we invite you to submit a revised version of the manuscript that addresses the points raised during the review process.

Please submit your revised manuscript by Sep 25 2026 11:59PM. If you will need more time than this to complete your revisions, please reply to this message or contact the journal office at plosone@plos.org. When you're ready to submit your revision, log on to https://www.editorialmanager.com/pone/ and select the 'Submissions Needing Revision' folder to locate your manuscript file.

- A letter that responds to each point raised by the academic editor and reviewer(s). You should upload this letter as a separate file labeled 'Response to Reviewers'.
- A marked-up copy of your manuscript that highlights changes made to the original version. You should upload this as a separate file labeled 'Revised Manuscript with Track Changes'.
- An unmarked version of your revised paper without tracked changes. You should upload this as a separate file labeled 'Manuscript'.

If you would like to make changes to your financial disclosure, please include your updated statement in your cover letter. Guidelines for resubmitting your figure files are available below the reviewer comments at the end of this letter.

If applicable, we recommend that you deposit your laboratory protocols in protocols.io to enhance the reproducibility of your results. Protocols.io assigns your protocol its own identifier (DOI) so that it can be cited independently in the future. For instructions see: https://journals.plos.org/plosone/s/submission-guidelines#loc-laboratory-protocols. Additionally, PLOS ONE offers an option for publishing peer-reviewed Lab Protocol articles, which describe protocols hosted on protocols.io. Read more information on sharing protocols at https://plos.org/protocols?utm_medium=editorial-email&utm_source=authorletters&utm_campaign=protocols.

As the corresponding author, your ORCID iD is verified in the submission system and will appear in the published article. PLOS supports the use of ORCID, and we encourage all coauthors to register for an ORCID iD and use it as well. Please encourage your coauthors to verify their ORCID iD within the submission system before final acceptance, as unverified ORCID iDs will not appear in the published article. *Only*  the individual author can complete the verification step; PLOS staff *cannot*  verify ORCID iDs on behalf of authors.

We look forward to receiving your revised manuscript.

Kind regards,

De-Chih Lee, Ph.D.

Academic Editor

PLOS One

Journal Requirements:

When submitting your revision, we need you to address these additional requirements.

1.Please ensure that your manuscript meets PLOS ONE's style requirements, including those for file naming. The PLOS ONE style templates can be found at

https://journals.plos.org/plosone/s/file?id=wjVg/PLOSOne_formatting_sample_main_body.pdf and

https://journals.plos.org/plosone/s/file?id=ba62/PLOSOne_formatting_sample_title_authors_affiliations.pdf

2. In the online submission form you indicate that your data is not available for proprietary reasons and have provided a contact point for accessing this data. Please note that your current contact point is a co-author on this manuscript. According to our Data Policy, the contact point must not be an author on the manuscript and must be an institutional contact, ideally not an individual. Please revise your data statement to a non-author institutional point of contact, such as a data access or ethics committee, and send this to us via return email. Please also include contact information for the third party organization, and please include the full citation of where the data can be found.

3. Thank you for providing your underlying data as Supporting Information.

We note that the data set contains text or data that is not in English. Please note that PLOS is an English-language publisher, so we require data sets to be provided in English as well. Please upload an English-language version of your data set.

This will also allow us to determine if your data follows PLOS standards per our Data Availability policy here: https://journals.plos.org/plosone/s/data-availability

4. If the reviewer comments include a recommendation to cite specific previously published works, please review and evaluate these publications to determine whether they are relevant and should be cited. There is no requirement to cite these works unless the editor has indicated otherwise.

Additional Editor Comments:

Please make the main revisions based on the review comments.

[Note: HTML markup is below. Please do not edit.]

Reviewers' comments:

Reviewer's Responses to Questions

**Comments to the Author**

1. Is the manuscript technically sound, and do the data support the conclusions?

Reviewer #1: Yes

Reviewer #2: No

Reviewer #3: Partly

Reviewer #4: Partly

**********

2. Has the statistical analysis been performed appropriately and rigorously?

Reviewer #1: Yes

Reviewer #2: No

Reviewer #3: Yes

Reviewer #4: Yes

**********

3. Have the authors made all data underlying the findings in their manuscript fully available?

The PLOS Data policy

Reviewer #1: Yes

Reviewer #2: No

Reviewer #3: Yes

Reviewer #4: No

**********

4. Is the manuscript presented in an intelligible fashion and written in standard English?

Reviewer #1: Yes

Reviewer #2: Yes

Reviewer #3: Yes

Reviewer #4: No

**********

Reviewer #1: Dear Editor,

Thank you for inviting me to review this manuscript, which reports the translation and psychometric evaluation of the Persian version of the Self‑Regulated Learning Scale for Clinical Nursing Practice. The study addresses a relevant gap in nursing education, and the authors have followed a systematic approach including forward‑backward translation, content validity assessment, exploratory and confirmatory factor analyses, and reliability testing. However, several methodological and reporting issues need to be addressed before the manuscript can be accepted.

The sample size justification is adequate for the analyses performed, but the handling of the split‑sample design (EFA n=160, CFA n=200) is not clearly tied to the original sample size calculation. The authors state that they aimed for 360 participants to meet both EFA and CFA requirements, which is appropriate. However, they do not report how the 360 were randomly divided into the two subsamples. Random splitting is acceptable, but the method should be described (e.g., using a random number generator in SPSS). Without this description, the independence of the EFA and CFA samples cannot be fully verified.

The exploratory factor analysis used maximum likelihood extraction with Promax rotation, which is reasonable given the expected correlation among factors. However, the authors report using multiple criteria for factor retention (eigenvalue >1, scree plot, parallel analysis), but in the results section only eigenvalues and cumulative variance are presented. The parallel analysis results are not reported. Parallel analysis is generally more accurate than the eigenvalue >1 rule, so its omission is a notable gap. The authors should either provide the parallel analysis output (e.g., comparing real‑data eigenvalues to random‑data eigenvalues) or state clearly that parallel analysis was not performed despite being mentioned in the methods. If it was performed, the results must be reported.

The confirmatory factor analysis model fit is excellent, but the authors do not report the standardized factor loadings from the CFA model in the text or in a table. They provide a path diagram (Figure 1) that is not visible in the review file, so the reader cannot verify the magnitude of the loadings. The text only states that loadings ranged from 0.66 to 0.96. This range should be presented with item numbers to allow assessment of which items have lower loadings. Additionally, the critical ratios (C.R.) are reported for a few items (e.g., “from 5.698 to 21.485”), but these are derived from unstandardized estimates and standard errors. Reporting the standardized loadings with their standard errors or t‑values would be more informative.

The convergent validity results are strong, but the average variance extracted (AVE) for the multidimensional thinking factor is 0.839, which is very high. An AVE above 0.80 can sometimes indicate item redundancy, especially given that this factor has only two items. The authors should comment on whether the two items are conceptually distinct enough or whether the high AVE suggests that they are nearly identical. The item content for multidimensional thinking (items 13 and 14: “I would like to develop my way of thinking...” and “I wonder if there are other ways of thinking...”) appears to be related but not redundant. However, the high AVE should be noted.

The discriminant validity assessment uses multiple criteria (Fornell‑Larcker, HTMT). The HTMT values are all below 0.85, which is acceptable. However, the correlation between intrinsic motivation and effort control is 0.414, and between synthesized knowledge and effort control is 0.405. These are moderate correlations, but they are not problematic. The authors should report the actual HTMT values in a table rather than just stating that they are below 0.85. The provided Table 4 shows HTMT values (e.g., 0.372, 0.386, 0.414), which are actually the factor correlations, not HTMT. The HTMT is a ratio that corrects for attenuation, and it is typically higher than the raw correlation. The authors incorrectly labeled the off‑diagonal entries as “HTMT Matrix” but those numbers appear to be the raw factor correlations (since they match the correlations reported in the same row/column). The HTMT should be computed and reported separately, or the authors should clarify that they are reporting the Fornell‑Larcker correlations and not HTMT. This is a significant error.

The reliability analysis is thorough, including Cronbach’s alpha, McDonald’s omega, Coefficient H, and GLB. However, the test‑retest reliability sample is only 30 participants. While this is acceptable for a preliminary estimate, the confidence intervals for the ICCs are not reported in the text or table. Table 5 shows ICC values but no confidence intervals. The authors should provide the 95% confidence intervals for each ICC to allow assessment of precision.

The discussion compares the Persian version’s psychometric properties to the original Japanese and Turkish versions, claiming “superior psychometric properties.” This is an overstatement. Differences in fit indices and reliability coefficients across studies can be due to sample characteristics, cultural factors, or random variation. The Persian version is not inherently superior; it is simply a successful adaptation. The authors should rephrase these claims to be more neutral, for example, “the Persian version demonstrated strong psychometric properties, comparable to or slightly better than the original and Turkish versions, which may reflect differences in sample characteristics or cultural context.”

The manuscript also contains minor errors. In Table 2, the column headings are misaligned, and the “A” column (Number agreeing on good relevance) appears to be repeated. The table should be reformatted for clarity. Additionally, the authors state that they used “IBM SPSS AMOS Graphics (Version 24; IBM Corp.)” but the current version of AMOS is 26 or higher; this is not a major issue but should be updated if possible.

Finally, the authors did not report the descriptive statistics for the scale items (means, standard deviations, skewness, kurtosis) beyond the factor means in Table 3. It would be helpful to include item‑level descriptive statistics in a supplementary table to assess floor/ceiling effects and normality.

Given these issues, the manuscript requires revision to correct the discriminant validity reporting error, provide parallel analysis results, report CFA standardized loadings, add ICC confidence intervals, and tone down the claims of superiority. The core validation is sound, and the scale appears to be a useful instrument. I therefore recommend minor revision. After addressing these points, the manuscript should be acceptable for publication.

Reviewer #2: The authors present a cross-cultural adaptation and psychometric evaluation of the Persian version of the Self-Regulated Learning Scale for Clinical Nursing Practice. The topic is relevant to nursing education, and the use of separate exploratory and confirmatory samples is a strength. The manuscript also assesses several measurement properties, including content validity, structural validity, internal consistency, discriminant validity, and test–retest reliability.

However, major methodological and reporting issues currently prevent a reliable interpretation of the findings. In particular, there appears to be a serious numerical inconsistency in the exploratory factor analysis. These concerns should be addressed before the manuscript can be considered technically sound.

Major comments

1. Major inconsistency in the EFA variance results

The manuscript states that the five factors explain 63.5% of the total variance. However, the percentages presented in Table 3 are:

Intrinsic motivation: 19.48%

Achievement motivation: 17.94%

Synthesized knowledge and nursing skills: 15.70%

Multidimensional thinking: 14.63%

Effort control: 13.74%

These values sum to approximately 81.49%, not 63.5%.

Similarly, the reported eigenvalues are 3.117, 2.871, 2.512, 2.341, and 2.198. Their sum is 13.039, which, when divided by 16 items, also corresponds to approximately 81.5% of variance. Therefore, the reported 63.5% appears inconsistent with Table 3.

This is a major issue because the percentage of explained variance is a central result reported in the Abstract, Results, Discussion, and Conclusion. The authors should verify the original output and correct the eigenvalues, percentages, or total variance explained throughout the manuscript. A complete EFA output or supplementary analysis file would assist verification.

2. EFA reporting is incomplete

The Methods state that eigenvalues, the scree plot, and parallel analysis were used to determine the number of factors. Nevertheless, the Results provide no parallel-analysis values, observed-versus-random eigenvalues, or scree-plot information.

Please report:

The number of factors suggested by parallel analysis.

The observed and simulated eigenvalues.

Whether all three retention methods supported five factors.

The factor-correlation matrix resulting from the Promax rotation.

The complete pattern matrix, including cross-loadings rather than only the intended loading of each item.

The extraction convergence and number of iterations.

The wording “items with cross-loadings below 0.40” is also unclear. Presumably, the authors mean that items were retained when their primary loading was at least 0.50 and all secondary loadings were below 0.40. This should be rewritten precisely.

3. Treatment of ordinal Likert data

The scale contains 16 items measured using a five-point Likert response format. The authors used maximum-likelihood estimation in both EFA and CFA, but they do not report whether univariate or multivariate normality assumptions were evaluated.

Please report item distributions, including skewness and kurtosis, and explain why conventional maximum likelihood was considered appropriate. If the items were substantially non-normal or ordinal assumptions were important, the authors should consider an ordinal-data method based on polychoric correlations and a robust estimator, such as WLSMV or robust maximum likelihood. A sensitivity analysis comparing the current model with an ordinal or robust model would strengthen the manuscript.

4. CFA model specification and possible model modification

The CFA produces exceptionally strong fit indices, including CFI = 0.980 and RMSEA = 0.044. However, the manuscript does not explain whether the model was specified strictly according to the original structure or whether modifications were made after examining modification indices.

Please clarify:

Whether correlated residuals were introduced.

Whether any parameters were added or removed after inspection of modification indices.

Whether all latent factors were allowed to correlate.

Whether the five-factor model was compared with plausible alternative models, such as a one-factor model, a two-factor model representing motivation and learning strategies, or a higher-order model.

Because the instrument is described as containing two broader subscales and five first-order factors, testing the hierarchical structure would be valuable. At minimum, the authors should explain why only the five-factor first-order model was tested.

The CFA results should also include the full 90% confidence interval for RMSEA rather than merely stating “RMSEA = 0.044 (90% CI).” Standardized residual information, such as SRMR, should be reported.

5. EFA and CFA sample allocation requires clarification

The authors report that 360 students were divided into EFA and CFA samples of 160 and 200 participants. Please explain exactly how participants were allocated to these two datasets. Was allocation random, chronological, or based on another procedure?

Because the groups differ in age, marital status, and academic-year composition, the authors should report whether the two subsamples were statistically comparable. The higher proportion of fourth-year students in the CFA group and the difference in mean age may affect the results.

The description of stratified random sampling also requires more detail. Please explain:

The sampling frame.

The number of eligible students in each academic year.

How students were selected within strata.

Whether computer-generated random numbers were used.

The number approached and responding within each stratum.

The reasons for non-participation among the 40 students who did not participate.

6. Test–retest reliability is inadequately reported

The Methods state that test–retest reliability was evaluated in 30 students after two weeks, but Table 5 is titled “n = 200.” The table therefore appears to incorrectly imply that the ICC estimates were calculated in 200 participants.

Please clearly distinguish the sample used for internal consistency from the sample used for test–retest reliability. Table 5 should specify n = 30 for ICC estimates.

The exact ICC model must also be reported, including:

One-way or two-way model.

Random-effects or mixed-effects model.

Consistency or absolute agreement.

Single-measure or average-measure ICC.

How the 30 retest participants were selected.

Whether their characteristics differed from those who were not retested.

The statement that ICC values above 0.75 indicate “excellent” reliability should also be revised. Common interpretation guidelines usually distinguish approximately 0.75–0.90 as good and values above 0.90 as excellent, depending on the context.

7. Internal consistency of multidimensional and two-item scales

The authors report Cronbach’s alpha and other reliability coefficients for the total 16-item score, even though the scale is explicitly multidimensional. A high total alpha does not by itself justify combining all items into one total score.

Please clarify whether the original instrument authorizes interpretation of a total score. If a total score is intended, evidence from a higher-order or bifactor model should be considered. Otherwise, reliability and interpretation should focus on the validated subscale or factor scores.

Two factors—multidimensional thinking and effort control—contain only two items each. Cronbach’s alpha is not particularly informative for two-item factors. The authors should report the inter-item correlation or Spearman–Brown coefficient and discuss the limited content coverage and stability of two-item constructs.

The manuscript should also explain how McDonald’s omega, coefficient H, and GLB were calculated, including the software or package used. The Statistical Analysis section currently states that SPSS and AMOS were used, but it is unclear how all these coefficients, particularly GLB and HTMT, were derived.

8. Discriminant-validity table requires correction and clarification

Table 4 is described as presenting CR, AVE, MSV, ASV, and an HTMT matrix. However, the diagonal values are shown as 1.000. If this is an HTMT matrix, the diagonal should generally be omitted or clearly identified. If it is intended as a Fornell–Larcker matrix, the diagonal should contain the square root of AVE rather than 1.000.

The Methods state that three approaches were used:

Fornell–Larcker criterion.

AVE compared with MSV and ASV.

HTMT.

However, the table does not clearly present the Fornell–Larcker matrix. Please present the relevant information in separate, clearly labelled sections or tables. It should also be clarified whether the off-diagonal values are latent correlations or HTMT ratios.

Bootstrapped confidence intervals for HTMT would provide stronger evidence of discriminant validity than relying only on a fixed threshold.

9. COSMIN claims should be moderated

The manuscript repeatedly states that the study followed COSMIN guidelines and describes its methods as fully aligned with COSMIN domains. However, not all relevant measurement properties were evaluated. For example, measurement error, hypothesis testing against related constructs, responsiveness, and criterion-related evidence were not assessed.

Furthermore, COSMIN content-validity assessment emphasizes relevance, comprehensiveness, and comprehensibility from both professionals and the target population. The manuscript should explain more fully how comprehensiveness was evaluated and provide the qualitative findings rather than only stating that minor modifications were made.

The wording should therefore be changed from broad claims of comprehensive COSMIN adherence to a more precise statement that selected measurement properties were evaluated with reference to COSMIN recommendations.

10. Content-validity procedures require additional reporting

The authors report I-CVI and modified kappa values but do not report a scale-level content validity index, such as S-CVI/Ave or S-CVI/UA. Please provide an appropriate scale-level measure.

The characteristics of the 10 experts should also be described, including their fields, academic qualifications, years of experience, and selection criteria.

For the qualitative face- and content-validity phases, please report examples of the changes made to individual items. The Discussion later refers to “cognitive interviews and multiple pilot tests,” but these procedures are not clearly described in the Methods. Either provide the corresponding methodology and results or remove this claim.

11. Additional validity evidence is limited

The authors assess convergent validity using AVE calculated from the same CFA model. This demonstrates convergence of items within each latent factor but does not establish convergence with an external measure of self-regulated learning or a theoretically related construct.

The manuscript should avoid implying that external convergent or criterion validity has been established. The lack of comparison with another validated measure should be acknowledged as a limitation.

12. Data availability does not appear to satisfy the journal’s stated policy

The Data Availability Statement indicates only that datasets are available from the corresponding author upon reasonable request. No ethical, legal, or privacy restriction is described.

The authors should deposit the de-identified minimum dataset underlying all reported results, along with the variable codebook and analysis syntax, in an appropriate public repository. If public sharing is prohibited, the authors should specify the exact ethical or legal restriction, identify the responsible data-access body, and provide a non-author institutional contact through whom qualified researchers can request access.

13. Participant flow and missing data

The manuscript states that cases with more than 10% missing data were excluded but does not report:

How many participants had missing responses.

Whether any participants were excluded for this reason.

How item-level missing values of 10% or less were handled.

Whether complete-case analysis, mean substitution, or another method was used.

Whether the 360 participants represent all complete cases.

A participant-flow description should be included.

14. Interpretation and generalizability are overstated

The sample was drawn from one nursing school at one university. Therefore, the conclusion that the instrument is suitable for “Persian-speaking nursing students” is broader than the evidence supports. The findings primarily support its use among undergraduate nursing students at the participating institution.

Claims that the scale can improve clinical competencies, curriculum development, teaching methodologies, or patient care are not directly tested by this cross-sectional validation study. These statements should be presented as possible future applications rather than demonstrated outcomes.

Minor comments

Please report the complete RMSEA 90% confidence interval.

Report TLI and SRMR in the Results, as these were mentioned or expected in the analytical framework.

Provide descriptive statistics for individual item response distributions, including floor and ceiling effects.

Consider reporting confidence intervals for alpha, omega, and other reliability estimates.

Correct the title of Table 5 to distinguish the CFA/internal-consistency sample from the 30-person retest sample.

In Table 5, one confidence interval appears as “0.765–884”; this likely requires correction to “0.765–0.884.”

Check the ordering and alignment of α, ω, H, and GLB headings in Table 5.

The pooled GPA standard deviation should be verified because it appears inconsistent with the subgroup standard deviations.

Replace statements such as “superior reliability” with neutral descriptions. Higher reliability coefficients do not necessarily indicate a superior instrument and may sometimes reflect item redundancy or sample-specific effects.

“All values exceeded 0.70” is written in the Methods as though it were a result. Move this statement to the Results.

Correct capitalization and grammar, including “Furthermore, The HTMT ratios” and “all analysis,” which should be “all analyses.”

Ensure consistent terminology for “factors,” “subscales,” and “latent factors.”

The Persian version of the final questionnaire should be included as supporting information, together with an English back-translation where permitted by the copyright holder.

The manuscript would benefit from professional English-language editing to remove repetitive and promotional wording.

Reviewer #3: This manuscript describes the translation and psychometric evaluation of the Persian version of the Self-Regulated Learning Scale in Clinical Nursing Practice (SRLS-CNP-P). The importance of self-regulated learning for nursing students and the relevance of cultural adaptions are described The study addresses a relevant topic in nursing education and follows established procedures for instrument adaptation and validation. The psychometric analyses appear to have been conducted appropriately and generally support the reliability and structural validity of the Persian version.

While I consider the methodological work to be sound, I have several concerns regarding the conceptual framing and interpretation of the construct being measured.

1. Theoretical grounding of self-regulated learning

The theoretical discussion of self-regulated learning (SRL) in the manuscript remains rather limited. Although Zimmerman and Pintrich are mentioned, the authors do not clearly specify which SRL framework underpins the instrument or how the five factors relate to established SRL theory.

More importantly, the scale appears to capture selected motivational and strategic aspects of learning rather than the broader SRL construct described in many contemporary models. Core components such as planning, monitoring, self-evaluation and regulation based on feedback are largely absent. A more explicit discussion of what aspect of SRL is actually being measured would strengthen the manuscript considerably. Previous reviews reviews of SRL theory have emphasized the multidimensional nature of SRL, encompassing cognitive, metacognitive, motivational, behavioral, and increasingly social processes (Panadero, 2017; Zimmerman, 2002).

2. Cultural adaptation

The translation process was carefully conducted and provides good evidence for linguistic equivalence. However, the manuscript occasionally seems to use the terms linguistic adaptation and cultural adaptation interchangeably.

The presented findings demonstrate that the Persian version is understandable and psychometrically stable, but they do not fully establish cultural equivalence of the underlying construct. Given that the instrument originated in Japan, a more critical discussion of potential cultural differences in learning, achievement motivation and professional socialization would be helpful. At present, the paper demonstrates successful translation and validation, but less evidence is provided regarding cross-cultural comparability of the construct itself.

3. Discriminant validity

The reported AVE, MSV, ASV and HTMT results indicate that the five factors are distinguishable from one another within the scale. However, this represents only a relatively restricted form of discriminant validity, as all analyses are based on factors derived from the same instrument. The study does not address whether the SRLS-CNP-P can be distinguished from related constructs such as self-efficacy, metacognitive regulation etc. Including external measures of related constructs would have provided a more convincing test of construct validity. As currently presented, the findings support factorial distinctiveness rather than broader discriminant validity.

4. Normative assumptions

A point I want to adress, concerns the positive framing of self-regulated learning throughout the manuscript. SRL is consistently presented as a desirable attribute associated with academic success, clinical competence, professional development and lifelong learning. Although this perspective is common in the literature, it risks turning SRL from an analytical construct into a normative ideal. In several places, the discussion appears to imply that improved learning outcomes are primarily a function of individual self-regulation. Factors such as supervision quality, characteristics of the clinical learning environment, institutional support receive much less attention.

A more balanced discussion acknowledging the interaction between individual and contextual influences would strengthen the argument.

Some items seem to reflect broader expectations associated with being a "good" nursing student rather than self-regulated learning specifically. For example, items emphasizing effort, persistence, independent practice and achievement may capture aspects of professional socialization or conformity with educational expectations. It may therefore be useful for the authors discuss that high scores in the scales could partly reflect alignment with professional norms rather than self-regulation alone.

5. Gender and Social Context

The study sample is predominantly female (66.7%) and unmarried (86.1%). Although this may reflect the demographic composition of nursing programs, the manuscript does not consider whether SRL may be shaped by gendered professional expectations, social roles, or broader cultural factors.

A brief discussion of these contextual influences would strengthen the interpretation of the findings and support the claim of cultural relevance.

Minor Comment

The discussion repeatedly suggests that the Persian version demonstrates “superior” psychometric performance compared with previous adaptations. This conclusion should be expressed more cautiously. Higher reliability coefficients or fit indices do not necessarily imply greater construct validity or theoretical superiority. A formulation emphasizing comparability with previous validation studies would be preferable.

In sum, the psychometric evidence presented is generally strong and supports the use of the Persian SRLS-CNP-P. However, the manuscript would benefit from a stronger theoretical discussion of self-regulated learning, a more nuanced consideration of cultural adaptation, and a clearer acknowledgement of the normative assumptions embedded in both the SRL construct and nursing education. These additions would substantially enhance the manuscript's conceptual rigor without requiring major additional analyses.

Reviewer #4: Overall, this manuscript presents a technically competent psychometric validation study that addresses a worthwhile methodological gap by adapting and validating the Persian version of the Self-Regulated Learning Scale in Clinical Nursing Practice. The study appears to have been conducted appropriately, and the reported findings generally support the authors' primary conclusions. However, I do not believe the manuscript is yet ready for publication in its current form.

The principal issues relate to the transparency and reporting of the psychometric analyses rather than the underlying study itself. In particular, the authors should justify their use of maximum likelihood estimation for ordinal Likert-scale data, report the results of the parallel analysis used for factor retention, clarify whether any confirmatory factor analysis model modifications were undertaken, and provide greater detail regarding statistical assumptions and missing data handling. The discussion also overstates the comparative superiority of the Persian version relative to previous adaptations and would benefit from a more balanced interpretation of the findings. Furthermore, the manuscript would be strengthened by reducing repetition in the discussion, improving alignment with COSMIN terminology, and ensuring compliance with PLOS ONE's data-sharing requirements through deposition of the anonymised dataset in a recognised public repository where possible.

Overall, I believe the manuscript has sufficient merit for publication following major revision, provided these reporting and interpretative issues are adequately addressed.

**********

what does this mean?). If published, this will include your full peer review and any attached files.

If you choose “no”, your identity will remain anonymous but your review may still be made public.

**Do you want your identity to be public for this peer review?**  For information about this choice, including consent withdrawal, please see our Privacy Policy

Reviewer #1:**Yes:** Hamid Sharif-Nia

Reviewer #2:**Yes:** Taimoor Asghar

Reviewer #3: No

Reviewer #4: No

**********

[NOTE: If reviewer comments were submitted as an attachment file, they will be attached to this email and accessible via the submission site. Please log into your account, locate the manuscript record, and check for the action link "View Attachments". If this link does not appear, there are no attachment files.]

To ensure your figures meet our technical requirements, please review our figure guidelines: https://journals.plos.org/plosone/s/figures

You may also use PLOS’s free figure tool, NAAS, to help you prepare publication quality figures: https://journals.plos.org/plosone/s/figures#loc-tools-for-figure-preparation.

NAAS will assess whether your figures meet our technical requirements by comparing each figure against our figure specifications.

Attachment Submitted filename: Comments for authors.pdf

# Author comment: Author response to Decision Letter 1

**DOI:** 10.1371/journal.pone.0358790.r002

1 Aug 2026

PLOS One

Academic Editor

Dear Professor De-Chih Lee,

Thank you for considering the revised version of our manuscript entitled "Psychometric properties of the Persian version of the self-regulated learning scale in clinical nursing practice for nursing students: A methodological study." We appreciate the editor and reviewers for highlighting essential modifications required for the manuscript. Their insightful comments have been incredibly valuable in restructuring our work. We have carefully taken these suggestions into account. A detailed explanation of the changes made in response to the reviewers' feedback is provided point by point in the following pages, with all modifications highlighted in yellow within the manuscript. We sincerely hope that these revisions meet the criteria for making the manuscript acceptable for publication in PLOS One.

We are looking forward to hearing from you.

Attachment Submitted filename: Response to Reviewers.docx

# Peer review: Decision Letter 1

**DOI:** 10.1371/journal.pone.0358790.r003

**Authors:** De-Chih Lee

31 Aug 2026

Dear Dr. Nemati-Vakilabad,

Thank you for submitting your manuscript to PLOS One. After careful consideration, we feel that it has merit but does not fully meet PLOS One’s publication criteria as it currently stands. Therefore, we invite you to submit a revised version of the manuscript that addresses the points raised during the review process.

Please make minor revisions based on the reviewer's comments.

Please submit your revised manuscript by Sep 30 2026 11:59PM. If you will need more time than this to complete your revisions, please reply to this message or contact the journal office at plosone@plos.org. When you're ready to submit your revision, log on to https://www.editorialmanager.com/pone/ and select the 'Submissions Needing Revision' folder to locate your manuscript file.

- A letter that responds to each point raised by the academic editor and reviewer(s). You should upload this letter as a separate file labeled 'Response to Reviewers'.
- A marked-up copy of your manuscript that highlights changes made to the original version. You should upload this as a separate file labeled 'Revised Manuscript with Track Changes'.
- An unmarked version of your revised paper without tracked changes. You should upload this as a separate file labeled 'Manuscript'.

If you would like to make changes to your financial disclosure, please include your updated statement in your cover letter. Guidelines for resubmitting your figure files are available below the reviewer comments at the end of this letter.

If applicable, we recommend that you deposit your laboratory protocols in protocols.io to enhance the reproducibility of your results. Protocols.io assigns your protocol its own identifier (DOI) so that it can be cited independently in the future. For instructions see: https://journals.plos.org/plosone/s/submission-guidelines#loc-laboratory-protocols. Additionally, PLOS One offers an option for publishing peer-reviewed Lab Protocol articles, which describe protocols hosted on protocols.io. Read more information on sharing protocols at https://plos.org/protocols?utm_medium=editorial-email&utm_source=authorletters&utm_campaign=protocols.

As the corresponding author, your ORCID iD is verified in the submission system and will appear in the published article. PLOS supports the use of ORCID, and we encourage all coauthors to register for an ORCID iD and use it as well. Please encourage your coauthors to verify their ORCID iD within the submission system before final acceptance, as unverified ORCID iDs will not appear in the published article. *Only*  the individual author can complete the verification step; PLOS staff *cannot*  verify ORCID iDs on behalf of authors.

We look forward to receiving your revised manuscript.

Kind regards,

De-Chih Lee, Ph.D.

Academic Editor

PLOS One

Journal Requirements:

If the reviewer comments include a recommendation to cite specific previously published works, please review and evaluate these publications to determine whether they are relevant and should be cited. There is no requirement to cite these works unless the editor has indicated otherwise.

Please review your reference list to ensure that it is complete and correct. If you have cited papers that have been retracted, please include the rationale for doing so in the manuscript text, or remove these references and replace them with relevant current references. Any changes to the reference list should be mentioned in the rebuttal letter that accompanies your revised manuscript. If you need to cite a retracted article, indicate the article’s retracted status in the References list and also include a citation and full reference for the retraction notice.

[Note: HTML markup is below. Please do not edit.]

Reviewers' comments:

Reviewer's Responses to Questions

**Comments to the Author**

Reviewer #2: All comments have been addressed

Reviewer #5: All comments have been addressed

**********

2. Is the manuscript technically sound, and do the data support the conclusions?

Reviewer #2: Yes

Reviewer #5: Partly

**********

3. Has the statistical analysis been performed appropriately and rigorously?

Reviewer #2: Yes

Reviewer #5: No

**********

4. Have the authors made all data underlying the findings in their manuscript fully available?

The PLOS Data policy

Reviewer #2: Yes

Reviewer #5: Yes

**********

5. Is the manuscript presented in an intelligible fashion and written in standard English?

Reviewer #2: Yes

Reviewer #5: Yes

**********

Reviewer #2: Thank you for your thorough revision. The manuscript is substantially improved and nearly ready for acceptance. However, please address the following minor points in your final proof:

Scale-level content validity (S-CVI/UA): You report S-CVI/UA = 0.75, which falls below the conventional threshold of ≥0.80. Please either cite a source justifying this value for a new instrument or briefly acknowledge it as a limitation in the text.

Pooled GPA standard deviation: In Table 1, the EFA and CFA subgroups report SDs of 3.58 and 3.16, yet the total sample SD is reported as 3.75. Given the similar means, the pooled SD should be approximately 3.35. Please verify your calculation and correct this value, or provide the exact method used to derive 3.75.

Tracked-changes artifacts: Please ensure the final clean version removes all merge artifacts (e.g., "81.4963.5%" in the Abstract).

These require no re-analysis, but they do need correction or brief clarification before the manuscript can be accepted.

Reviewer #5: The revised manuscript has addressed most of the previous methodological and reporting concerns in a satisfactory manner. They also clarified that the EFA and CFA analyses were conducted on independent subsamples of 160 and 200 participants, respectively, which substantially improves the transparency of the psychometric design. My comments as followings:

Several minor but important issues. First, the content-validity reporting is still internally inconsistent. In the Methods, the manuscript states that S-CVI/UA should meet an acceptable threshold of ≥0.80, but the Results report S-CVI/UA = 0.75 and still describe the instrument as having strong overall content validity.

Second, the demographic table still appears to contain a numerical inconsistency. The EFA and CFA subgroups report GPA SDs of 3.58 and 3.16, but the total sample SD is reported as 3.75. Given the subgroup means and SDs, the pooled total SD should not normally exceed both subgroup SDs by this amount unless there is a specific calculation issue or data-distribution explanation.

Third, Table 5 contains a clear typographical or numerical error. The diagonal value for Achievement Motivation is reported as 1.847, although the square root of AVE for AVE = 0.718 should be approximately 0.847. This should be corrected, because it appears in a key validity table.

Fourth, the reliability table title still states n = 200, even though the table includes both internal-consistency estimates based on n = 200 and test–retest ICC estimates based on n = 30. The table itself labels the two sections, but the title should be revised to avoid the impression that ICCs were calculated from 200 participants.

Overall, the revised manuscript is substantially improved and the main psychometric evidence is now sufficiently transparent.

**********

what does this mean?). If published, this will include your full peer review and any attached files.

If you choose “no”, your identity will remain anonymous but your review may still be made public.

**Do you want your identity to be public for this peer review?**  For information about this choice, including consent withdrawal, please see our Privacy Policy

Reviewer #2:**Yes:** Taimoor Asghar

Reviewer #5: No

**********

[NOTE: If reviewer comments were submitted as an attachment file, they will be attached to this email and accessible via the submission site. Please log into your account, locate the manuscript record, and check for the action link "View Attachments". If this link does not appear, there are no attachment files.]

To ensure your figures meet our technical requirements, please review our figure guidelines: https://journals.plos.org/plosone/s/figures

You may also use PLOS’s free figure tool, NAAS, to help you prepare publication quality figures: https://journals.plos.org/plosone/s/figures#loc-tools-for-figure-preparation.

NAAS will assess whether your figures meet our technical requirements by comparing each figure against our figure specifications.

# Author comment: Author response to Decision Letter 2

**DOI:** 10.1371/journal.pone.0358790.r004

31 Aug 2026

We sincerely thank the reviewers for their thoughtful and constructive observations. Their careful attention to the accuracy of our reporting is greatly appreciated.

Attachment Submitted filename: Response_to_Reviewers_auresp_2.docx

# Peer review: Decision Letter 2

**DOI:** 10.1371/journal.pone.0358790.r005

**Authors:** De-Chih Lee

6 Sep 2026

Psychometric properties of the Persian version of the self-regulated learning scale in clinical nursing practice for nursing students: A methodological study

PONE-D-26-25065R2

Dear Dr. Nemati-Vakilabad,

We’re pleased to inform you that your manuscript has been judged scientifically suitable for publication and will be formally accepted for publication once it meets all outstanding technical requirements.

Within one week, you’ll receive an e-mail detailing the required amendments. When these have been addressed, you’ll receive a formal acceptance letter and your manuscript will be scheduled for publication.

An invoice will be generated when your article is formally accepted. Please note, if your institution has a publishing partnership with PLOS and your article meets the relevant criteria, all or part of your publication costs will be covered. Please make sure your user information is up-to-date by logging into Editorial Manager at Editorial Manager® and clicking the ‘Update My Information' link at the top of the page. For questions related to billing, please contact billing support.

If your institution or institutions have a press office, please notify them about your upcoming paper to help maximize its impact. If they’ll be preparing press materials, please inform our press team as soon as possible -- no later than 48 hours after receiving the formal acceptance. Your manuscript will remain under strict press embargo until 2 pm Eastern Time on the date of publication. For more information, please contact onepress@plos.org.

Kind regards,

De-Chih Lee, Ph.D.

Academic Editor

PLOS One

Additional Editor Comments (optional):

Reviewers' comments:

Reviewer's Responses to Questions

**Comments to the Author**

Reviewer #2: All comments have been addressed

Reviewer #5: All comments have been addressed

**********

2. Is the manuscript technically sound, and do the data support the conclusions?

Reviewer #2: Yes

Reviewer #5: Yes

**********

3. Has the statistical analysis been performed appropriately and rigorously?

Reviewer #2: Yes

Reviewer #5: Yes

**********

4. Have the authors made all data underlying the findings in their manuscript fully available?

The PLOS Data policy

Reviewer #2: Yes

Reviewer #5: Yes

**********

5. Is the manuscript presented in an intelligible fashion and written in standard English?

Reviewer #2: Yes

Reviewer #5: Yes

**********

Reviewer #2: This is much improved. However I re-checked the revision and found two reporting inconsistencies that need fixing before acceptance.

1. Content validity indices. The reported S-CVI/Ave (0.91) and S-CVI/UA (0.75) dont match the item-level I-CVI's shown in Table 2. With 10 experts and the pattern there (seven items at 0.8, six at 0.9, three at 1.0), the average is 0.875 - not 0.91. Also 0.91 is mathematically impossible with this expert/item structure anyway (average of fractions with denominator 10 must be a multiple of 0.00625, and 0.91 isnt). S-CVI/UA should be 0.188 (only 3 of 16 items had perfect agreement), not 0.75. Please re-calculate, correct the values, and revise the limitation discussion. If UA is really 0.19 thats a considerably weaker result than 0.75 and needs to be framed appropriately.

2. Table 4. Item 7 (Achievement Motivation) is misplaced under Synthesized Knowledge, and Item 9 is missing from the table entirely. Please fix the factor assignments and add Item 9's loading, S.E., and C.R.

Fix these and the manuscript is acceptable. The WLSMV sensitivity analysis, competing model testing, and the Discussion are real strengths of this work.

Reviewer #5: The authors have adequately addressed the remaining numerical and reporting issues raised in the previous round. The corrected values are now consistent with the manuscript’s psychometric analysis, and the related descriptions have been revised accordingly. This manuscript is also sufficiently readable in its current form.

**********

what does this mean?). If published, this will include your full peer review and any attached files.

If you choose “no”, your identity will remain anonymous but your review may still be made public.

**Do you want your identity to be public for this peer review?**  For information about this choice, including consent withdrawal, please see our Privacy Policy

Reviewer #2:**Yes:** Taimoor Asghar

Reviewer #5: No

**********

# Editor report: Acceptance letter

**DOI:** 10.1371/journal.pone.0358790.r006

**Authors:** De-Chih Lee

PONE-D-26-25065R2

PLOS One

Dear Dr. Nemati-Vakilabad,

I'm pleased to inform you that your manuscript has been deemed suitable for publication in PLOS One. Congratulations! Your manuscript is now being handed over to our production team.

At this stage, our production department will prepare your paper for publication. This includes ensuring the following:

* All references, tables, and figures are properly cited

* All relevant supporting information is included in the manuscript submission,

* There are no issues that prevent the paper from being properly typeset

You will receive further instructions from the production team, including instructions on how to review your proof when it is ready. Please keep in mind that we are working through a large volume of accepted articles, so please give us a few days to review your paper and let you know the next and final steps.

Lastly, if your institution or institutions have a press office, please let them know about your upcoming paper now to help maximize its impact. If they'll be preparing press materials, please inform our press team within the next 48 hours. Your manuscript will remain under strict press embargo until 2 pm Eastern Time on the date of publication. For more information, please contact onepress@plos.org.

You will receive an invoice from PLOS for your publication fee after your manuscript has reached the completed accept phase. If you receive an email requesting payment before acceptance or for any other service, this may be a phishing scheme. Learn how to identify phishing emails and protect your accounts at https://explore.plos.org/phishing.

If we can help with anything else, please email us at customercare@plos.org.

Thank you for submitting your work to PLOS One and supporting open access.

Kind regards,

PLOS One Editorial Office Staff

on behalf of

Dr. De-Chih Lee

Academic Editor

PLOS One
